# Functional and Trophic Structuring of Micronektonic Fishes in the Northeast Atlantic

**DOI:** 10.1002/ece3.74275

**Published:** 2026-09-10

**Authors:** Hélène Thibault, Frédéric Ménard, Gildas Roudaut, Anne Lebourges‐Dhaussy, Yves Cherel, Alejandro Ariza, Leandro Nolé Eduardo, Séverine Martini

**Affiliations:** ^1^ Aix Marseille Univ, Université de Toulon CNRS, IRD, MIO Marseille France; ^2^ Department of Biological Sciences University of Bergen Bergen Norway; ^3^ LEMAR, UBO, CNRS, IRD, Ifremer Plouzané France; ^4^ CEBC, UMR 7372 du CNRS‐La Rochelle Université Villiers‐en‐Bois France; ^5^ DECOD, Ifremer, INRAE, Institut Agro Nantes France; ^6^ MARBEC, Univ. Montpellier, CNRS, Ifremer, IRD Sète France

**Keywords:** functional indices, mesopelagic fish, mesopelagic zone, micronekton, stable isotopes, trait‐based approach, trophic partitioning

## Abstract

In pelagic ecosystems, sharp vertical gradients in resources and environmental conditions structure species adaptations, interactions and community assembly. Yet, the ecological mechanisms underlying these patterns remain poorly resolved. To investigate the functional and trophic structure of micronektonic fishes, specimens were collected across the epipelagic (0–200 m) and mesopelagic layers (200–1300 m) using a midwater trawl. A functional analysis of 85 species, characterized by 10 categorical traits, was conducted to define functional groups, identify key ecological trade‐offs among them and characterize the functional structure across depth strata. Stable carbon and nitrogen isotope values of fish tissues provided complementary insights into trophic structure, linking functional diversity with patterns of resource partitioning along the vertical gradient. We identified four functional groups whose trade‐offs reflect metabolic adaptations primarily shaped by diet. These adaptations enable species to cope with pressure and light gradients while enhancing resource acquisition and predator avoidance, particularly in upper layers. Communities showed a clear vertical transition from migratory, surface‐linked zooplanktivores in upper layers to resident, deeper‐dwelling assemblages with more diverse feeding guilds associated with larger body sizes and increased reliance on alternative carbon pathways. Consistent with ecological competition theory, deeper layers exhibited high functional and taxonomic diversity under food limitation, whereas food‐rich upper layers showed lower functional diversity and greater ecological similarity. Overall, vertical partitioning and functional specialization jointly structure mesopelagic communities, shaping trophic pathways, resilience and contributions to carbon cycling in pelagic ecosystems.

## Graphical abstract

Sharp vertical gradients structure the functional and trophic organization of mesopelagic micronektonic fishes, driving a transition from migratory, surface‐linked assemblages in upper layers (0–700 m) to resident communities adapted to deep, low‐light environments (> 700 m). Deep mesopelagic assemblages exhibit high functional and trophic diversity, suggesting coexistence through resource specialization across distinct carbon pathways. These patterns highlight the importance of vertical partitioning and functional specialization in shaping trophic pathways, community resilience and carbon cycling in pelagic ecosystems.

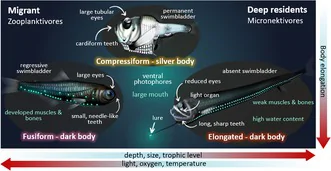

## 1 Introduction

Micronekton comprises small, actively swimming organisms, typically smaller than 20 cm, including several taxonomic groups. In this study, we focus on micronektonic fishes from the midwater environment, which are most abundant between the surface and 1000 m depth, spanning both the epipelagic (0–200 m) and mesopelagic (200–1000 m) zones (e.g., Angel and de C Baker 1982). Also called the twilight zone, its lower boundary lies within the ocean's sunless depths, above which sunlight still remains. This vertical habitat is shaped by strong gradients in light, temperature, pressure, oxygen availability and nutrient concentrations, with spatial and temporal stability increasing with depth layers (Robinson et al. 2010). The vertical dimension of the pelagic habitat was found to be the primary factor in structuring the composition of pelagic communities for both plankton and micronekton, rather than horizontal variability (Longhurst 1985; Sutton et al. 2008).

Mesopelagic fishes, living below the epipelagic zone, represent one of the largest reservoirs of vertebrate biomass in the ocean (Irigoien et al. 2014). Since the ‘azoic theory’ (Forbes 1844), 150 years of deep‐sea exploration have revealed the exceptional diversity of mesopelagic fishes, while many species still remain undescribed (Webb et al. 2010). These organisms exhibit various physiological, morphological and behavioral adaptations (Eduardo et al. 2024), enabling niche exploitation across depth strata. They serve as critical trophic intermediaries, linking surface production to higher predators (Drazen and Sutton 2017; Iglesias et al. 2023), depending on the organic matter flux from surface waters and predator–prey interactions. Through diel‐modulated niche adaptation, vertical migrants can cross contrasting conditions twice daily, with depth‐stratified migrant communities structured as a ladder of migration (Vinogradov 1962), enhancing active carbon and nutrient transport at depth and reinforcing trophic connections within the mesopelagic ecosystem (Eduardo et al. 2024; Sutton et al. 2013). Despite increasing recognition of their ecological significance (e.g., Saba et al. 2021), the drivers of mesopelagic fishes' diversity and their implications for ecosystem functioning and services remain poorly understood (Martin et al. 2020).

Trait‐based approaches have been increasingly used to describe the complexity of marine ecosystem functioning and to address the processes that structure their diversity (Kirboe et al. 2018; Martini et al. 2021). Individuals are characterized by essential traits related to their fitness, including survival, feeding and reproduction. The functional diversity within different environments helps to capture the ecological roles and functions of individuals in ecosystems, providing key insights into ecological niches and adaptive strategies (Petchey and Gaston 2006; Mouillot et al. 2013). In fact, the distribution of functional traits can help to understand the nature and strength of organisms' contributions to ecosystem processes (De Bello et al. 2010), such as nutrient cycling and carbon export. Regarding micronektonic fishes, particularly in the deep ocean, trade‐offs and interrelationships between traits are known for only a small number of species (Villeger et al. 2017). Mesopelagic fishes are often treated as a single ecological group or categorized by a single vertical migration behavior in models of carbon flux, due to the limited information available to accurately define their traits (Davison et al. 2013; Saba et al. 2021; Ariza et al. 2015). Yet, these fishes display remarkable morphological diversity, pronounced trophic specialization and considerable variability in both vertical migration behavior and spatial distribution (Hopkins et al. 1996; Eduardo et al. 2024). Functional indices are useful tools to address community assembly rules and their roles in ecosystem processes, considering both the distribution of species traits and their abundance in a community (Villeger 2008; Mouillot et al. 2013). Niche partitioning is reflected in the degree of morphological divergence among species exploiting the same resource dimension (Hutchinson 1959). Despite the ecological importance of these functional adaptations, functional diversity remains much less studied than taxonomic diversity in mesopelagic fishes. Trait‐based approaches have been applied to mesopelagic fish assemblages, focusing on morphological patterns associated with ecological strategies (Tuset et al. 2014), functional niche partitioning (Kumar et al. 2017) and functional diversity across depth layers (Aparecido et al. 2023; Loutrage et al. 2025). Although vertical distribution and resource partitioning are recognized as key drivers of ecological structure in mesopelagic communities, they remain difficult to characterize due to the high diversity of species, their dynamic vertical movements and the limited ecological information available for most taxa.

Moreover, functional trait analyzes can be strengthened by integrating complementary ecological tracers, yielding a more holistic representation of ecosystem organization and energy flow. Behavioral and trophic ecology are often analyzed separately for fishes (Luiz et al. 2019), but combining these approaches allows for the evaluation of how pelagic species differentiate ecological strategies and partition resources. For instance, carbon and nitrogen stable isotope ratios in fish tissues ($\delta^{13}$C and $\delta^{15}$N) provide time‐integrated proxies of resource use and trophic position, refining inferences on foraging dynamics and food‐web structure (e.g., Cherel et al. 2010; Fanelli et al. 2011; Menard et al. 2014; Annasawmy et al. 2020). Specifically, $\delta^{13}$C reflects primary carbon sources and spatial foraging origin (e.g., epipelagic vs. mesopelagic), whereas $\delta^{15}$N values in mesopelagic species can reflect multiple ecological dimensions, including trophic position and feeding depth and regional variation in nitrogen baselines (Post 2002; Gloeckler et al. 2018; Eduardo et al. 2023). Integrating these trophic proxies with functional trait analyzes can enable a more nuanced characterization of realized niches, quantification of trophic redundancy or complementarity and a better understanding of the mechanisms facilitating mesopelagic species coexistence and energy transfer in the water column.

The present study is based on trawl data collected during the APERO scientific cruise in the Northeast Atlantic. We investigated the functional diversity of micronektonic fishes, including morphological, behavioral and trophic features, with the aim of better understanding how these species are distributed along vertical gradients and how this contributes to pelagic ecosystem functioning. Specifically, we addressed: (i) How are mesopelagic communities structured along the water column? (ii) Which key traits and trade‐offs may explain the underlying processes driving fish functional diversity? (e.g., vertical behavior, diet, morphological characteristics) (iii) How do stable isotope values reflect trophic and functional redundancy or complementarity in mesopelagic communities? (iv) How is species coexistence shaped by vertical gradients within the pelagic environment?

These questions were investigated through an integrated approach combining functional trait analysis and stable isotope data to highlight key trade‐offs associated with differing resource‐use strategies and environmental adaptations, spanning from productive epipelagic waters to resource‐limited mesopelagic depths.

## 2 Material and Methods

### 2.1 Data Collection

**FIGURE 1:**
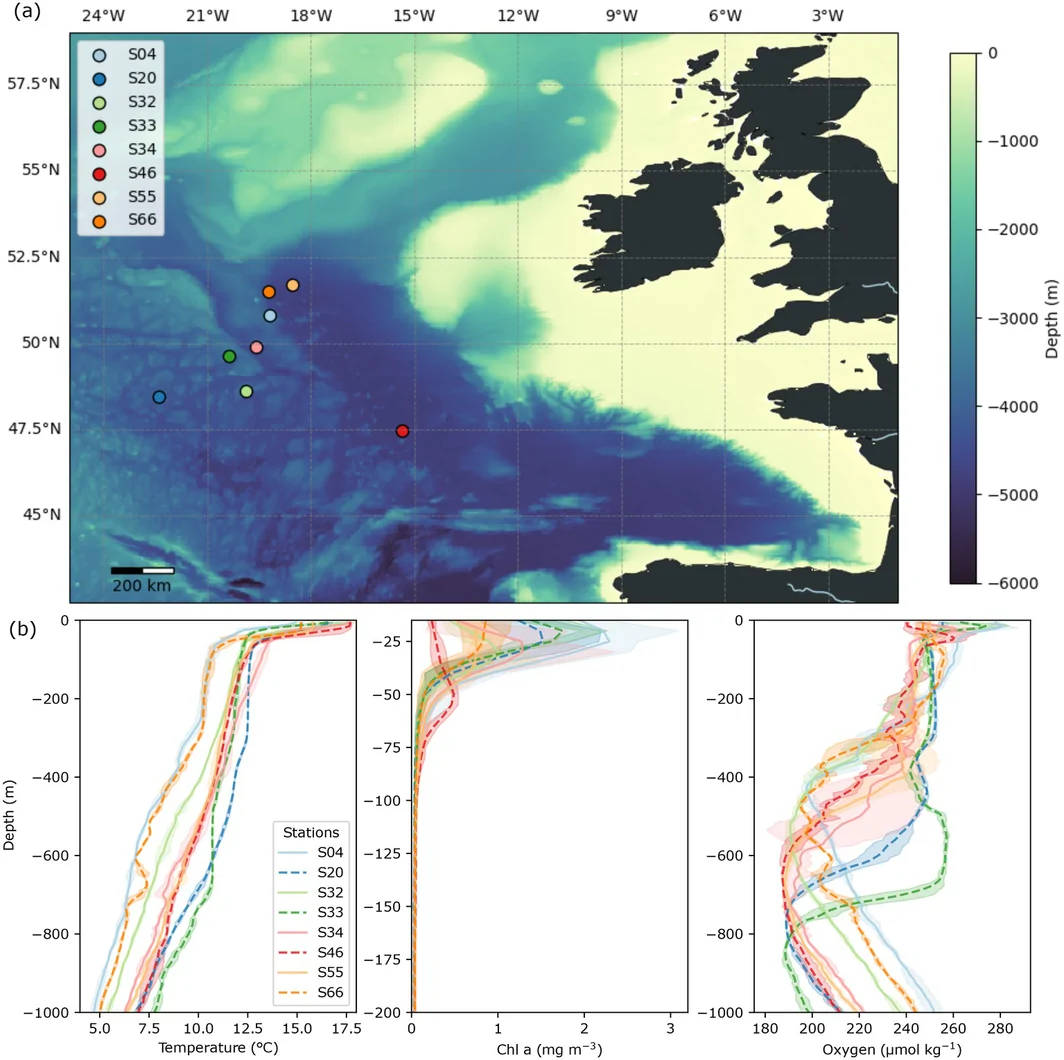
Map of survey area showing trawling stations (a) and CTD profiles for each station including average profile and standard deviation (b). A detailed description of the hauls deployed during the stations can be found in Table S1.

This study was conducted during the APERO oceanographic cruise (https://doi.org/10.17600/18000666) aboard the R/V *Thalassa* in the northeast Atlantic Ocean between June 3 and July 17, 2023 (Figure 1). The study area is characterized by a dynamic and productive open‐ocean environment. The survey was conducted under post‐bloom conditions across stations with different hydrographic conditions, likely representing different stages of the seasonal decline in primary production. At each station lasting more than 24 h, four vertical profiles of temperature, salinity, chlorophyll‐a fluorescence and oxygen concentrations were collected from the surface to at least 1000 m with a Seabird 911+ CTD (conductivity, temperature, depth) equipped with an oxygen sensor and a fluorescence probe.

**FIGURE 2:**
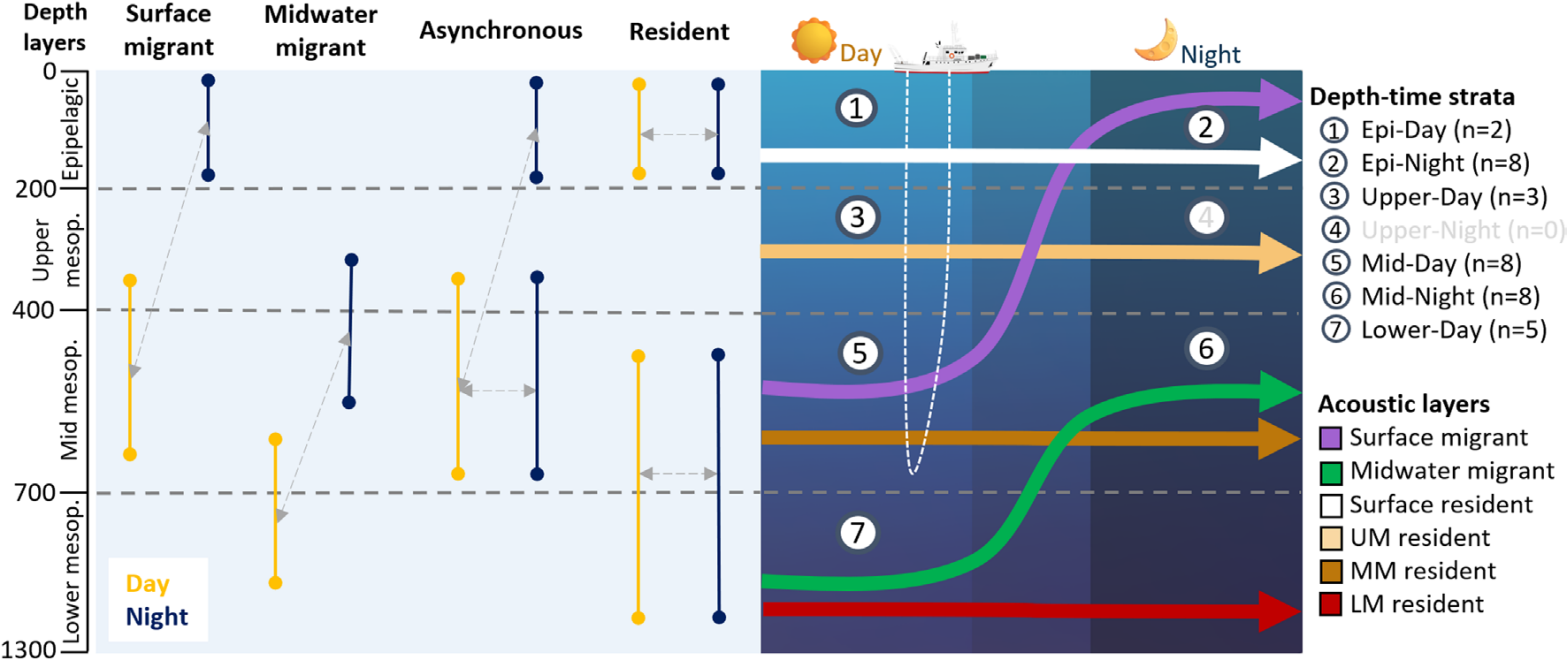
Conceptual diagram illustrating migration patterns of micronektonic fishes included in the functional analysis and detected acoustically. The dotted white line represents the trajectory of the trawl during descent, fishing and ascent, targeting specific acoustic layers while potentially sampling additional layers during retrieval. Commonly observed acoustic layers include surface and midwater migrants, as well as resident communities distributed across multiple depth strata (epipelagic, upper mesopelagic, mid‐mesopelagic and lower mesopelagic; see Figure S1). Depth strata are numbered from 1 to 7, each containing n samples corresponding to the number of stations sampled within that layer during daytime or nighttime. Details of the trawl hauls, including sampling depths, are provided in Table S1.

Micronektonic fishes were collected at eight long‐duration stations, including day and night sampling, using a midwater trawl (body mesh of 80 mm decreasing to cod‐end mesh of 10 mm, net mouth 24 × 24 m). The target depths for each sampling layer were defined based on acoustic detections using a Simrad EK80 split‐beam scientific echosounder (18 and 38 kHz). Distinct depth layers, ranging from the surface to 1300 m, were identified from the observed acoustic scattering layers and were sampled day and night at different depths. These layers indicate the positions and the daily movements of organisms in the water column (Figure S1). Different migration patterns could then be defined: surface migrant, midwater migrant, asynchronous migrant and resident, as illustrated in Figure 2. Each collected organism was identified at the species level, measured (standard length) and all individuals of the same species were weighed together.

Abundance estimates (ind m^−3^) were calculated from trawl sampling to compare the composition of micronektonic fish communities at different depth layers during the day and night. Each trawl haul was assigned to a depth‐time strata, that is, at each station and depth layer sampled during the day or night based on the targeted acoustic layers (Figure 2, Figure S1). However, due to net opening during retrieval, trawls may have also captured species from shallower, non‐targeted layers. To minimize this potential bias, particularly in deeper layers (i.e., mid‐ and lower mesopelagic), we excluded species that were also found in shallower trawls conducted at the same station and time period (day or night). An exception was found for three species where we observed different migrating patterns depending on size classes. These species were *Benthosema glaciale* , *Stomias boa* and *Chauliodus sloani* . For each trawl haul, the sampled volume (V in m^3^) was calculated to estimate community densities and biomass based on the estimated net mouth opening (S in m^2^) and the distance covered by the trawl (D in m) as V=SD, with $S=\frac{h}{2}\frac{v}{2}\pi$ and $D=2R\sin^{-1}\left(\sqrt{a}\right)$, where a is calculated as follows:

(1) $$a=\sin{\left(\frac{\mathrm{la}t_{2}-\mathrm{la}t_{1}}{2}\right)}^{2}+\cos\left(\mathrm{la}t_{1}\right)\cos\left(\mathrm{la}t_{2}\right)\sin{\left(\frac{\mathrm{lo}n_{2}-\mathrm{lo}n_{1}}{2}\right)}^{2}$$

where h and v are the net horizontal and vertical mouth openings (m); *R* = 6371 is the Earth's radius (km); and ${\mathrm{lat}}_{1}$, ${\mathrm{lat}}_{2}$, ${\mathrm{lon}}_{1}$, ${\mathrm{lon}}_{2}$ are the latitude and longitude of the start and end of fishing at the target depth layer (radian).

### 2.2 Data Analysis

#### 2.2.1 Functional Diversity

**FIGURE 3:**
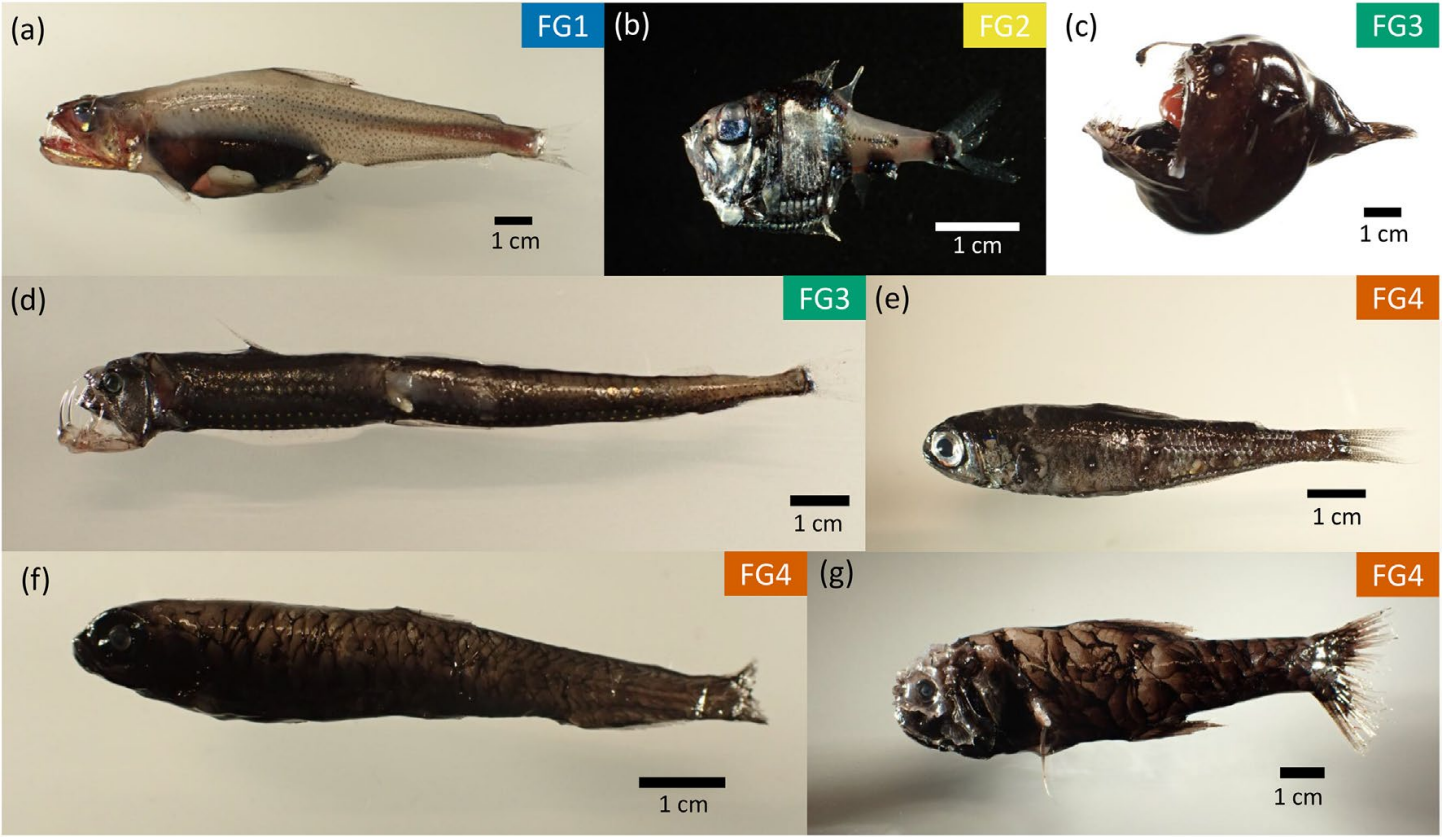
Pictures of mesopelagic fish species taken on board including *Evermanella balbo* (a), *Argyropelecus hemigymnus* (b), *Melanocetus johnsonii* (c), *Chauliodus sloani* (d), *Myctophum punctatum* (e), *Bathylagus euryops* (f) and *Scopelogadus beanii* (g). Species are associated with their functional group (FG).

The trait matrix was built on 10 fundamental traits of micronektonic fishes (Table 1; see Thibault (2026), including the trait matrix and references). All traits are categorical, mainly composed of morphological features, along with feeding guilds and migration patterns. 85 species of micronektonic fishes were selected, including all species for which at least 2 individuals were collected during the APERO cruise. Trait collection was based on the available literature, except for the ratio of eye diameter to total body length, for which measurements from the specimens' pictures taken on board were made using ImageJ (Abramoff et al. 2004; Figure 3). This ratio was then transformed into three size classes (low ratio: 0.002–0.026; med ratio: 0.029–0.052; high ratio: 0.055–0.140) using hierarchical agglomerative clustering based on Euclidean distance, and clusters were formed using Ward's minimum variance method (Husson et al. 2011; Legendre and Legendre 2012).

**TABLE 1:** Trait categories and descriptions used in the Multiple Correspondence Analysis.

Category	Trait	Description	Content
Morphological	Body shape	General body shape classification	Compressiform; fusiform; elongated; eel‐like
	Ratio eye/TL	Ratio of eye diameter to total body length	Small ratio; med ratio; high ratio
	Skin color	Main color of the skin	Black; brown; silver; light; orange
	Photophore	Presence of photophores	Presence (Ph1); absence (Ph0)
	Lure	Presence of a lure apparatus	Presence (lure)/absence (no lure)
	Teeth type	Patterns of dentition	No teeth; slender, sharp (sharp); numerous, needlelike (villiform); flat‐bladed, triangular (triangular); caniniform; cardiform
	Swim bladder	Presence of a swim bladder	Permanent (sb. perm); absent (sb. abs); regressive (sb. regress)
	Eye structure	Shape of the eye	Spherical; tubular
Physiological	Diet	Feeding guilds	Zooplanktivore; micronektivore; gelativore; generalist
Behavior	Migration pattern	Type of migration behavior	Surface migrant; midwater‐migrant; asynchronous; surface resident; deep resident (deep)

Trait selection was constrained by data availability for fish species in the literature and aimed to reduce redundancy among variables. Traits were defined as species level characteristics describing the most common features of the adult stage. An initial set of categorical traits was evaluated using Multiple Correspondence Analysis (MCA) to examine associations among trait categories and identify redundant information. Traits or categories showing strong associations and contributing similarly to the same MCA dimensions were considered redundant, and only representative traits were retained to avoid overrepresentation of specific functional characteristics in the functional space. Morphological traits were selected due to their links to key ecological functions such as resource acquisition, predator avoidance and growth (Andresen et al. 2025). Vertical distribution reflects feeding habitat and influences species' roles in nutrient transport across depth layers (Mouillot et al. 2014), while feeding guilds represent trophic interactions and resource use within the ecosystem. Vertical behavior was primarily assigned based on available literature; however, in cases where information was lacking or inconsistent, it was inferred from the observed patterns of depth occurrence between day and night in this study to ensure consistency with the sampled distribution (see Supporting Information for day and night distribution depths of each species).

Species sharing the same combination of traits were grouped into functional entities. The functional space was derived from the trait matrix using a MCA (Benzecri 1977; Husson et al. 2011) on categorical traits with the R package FactoMineR. MCA reduces the dimensionality of categorical trait data while preserving associations among trait modalities and functional entities. The proportion of inertia of the axes was assessed using Benzécri‐corrected eigenvalues. In order to define the functional space, the number of dimensions (MCA axes) was chosen based on the quality of the functional space, defined by the mean squared deviation index (mSD) which compares the trait‐based distance between functional entities and the distance in the functional space built using principal coordinates analysis, with the R package mFD (Magneville et al. 2022). Functional entity coordinates were computed along each MCA axis based on their combinations of trait values. The coordinates of each functional entity on the selected MCA axes were then used to compute the Euclidean distance matrix between the species. A summary of the functional analysis can be found in Figure S2.

Hierarchical agglomerative clustering analysis was performed on functional entities coordinates using the Unweighted Pair Group Method with Arithmetic Mean (UPGMA; Mouchet et al. 2008). This method follows an iterative process in which species are initially considered as individual clusters and are then progressively merged based on their average pairwise distances. This analysis produced a functional dendrogram representing the relationships among species. To define functional groups, the final dendrogram structure was used to determine appropriate cutting levels (Figure S4). The optimal clustering level was identified using the highest silhouette score, ensuring that species within each group were functionally similar while maximizing the distinction between groups.

We calculated four complementary Functional Diversity indices, ranging from 0 to 1, to optimally describe traits distribution across depth layers (epipelagic, upper‐mesopelagic, mid‐mesopelagic, lower‐mesopelagic, with depths represented in Figure S1) and between day and night for the 8 stations. Functional Richness (fRic) quantifies the extent of functional space occupied by functional entities (FEs) within a community. This metric quantifies the volume enclosed within the minimum convex hull, including all species within a community. Consequently, fRic is determined solely by the identity of the species, independent of their relative abundances and is primarily shaped by the most functionally extreme species that define the boundaries of the convex hull (Villeger et al. 2008). Functional Evenness (fEve) characterizes the homogeneity of FEs' abundance distribution along the minimum spanning tree that connects FEs in the multidimensional functional space. Functional Dispersion (fDis) represents the mean Euclidean distance of individual FEs from the centroid of all FEs within the trait space, weighted by species abundance. Functional Divergence (fDiv) quantifies the proportion of FEs' abundance concentrated with the most extreme functional traits, which define the outer limits of the functional space (Mouillot et al. 2013). More details about the computation of the functional index can be found in Villeger et al. (2008) and in Laliberte and Legendre (2010) for fDis.

#### 2.2.2 Stable Isotope Analysis

A total of 204 samples were collected and analyzed in four depth‐time strata (Table S2), namely the epipelagic layer during the night (Epi‐Night, *n* = 40, SR = 12), the mid‐mesopelagic layer during the day (Mid‐Day, *n* = 56, SR = 16) and night (Mid‐Night, *n* = 54, SR = 18) and the lower‐mesopelagic layer during the day (Lower‐Day, *n* = 54, SR = 18). The highest abundance and diversity were observed in the latter. For each individual, the standard length (mm) was measured on board, and a small piece of muscle tissue was collected and frozen at −20°C on board.

All muscle tissue samples were freeze‐dried for 48 h using a Christ Alpha 1–4 LSC freeze‐dryer and subsequently ground to a fine, homogeneous powder with an automatic ball mill (RETSCH MM200).

Stable carbon and nitrogen isotope ratios were determined with a Thermo Scientific FLASH 2000 elemental analyzer coupled to a Delta V Plus isotope ratio mass spectrometer (Pôle de Spectrométrie Océan, Plouzané, France). $\delta^{13}$C and $\delta^{15}$N values (‰) were calculated relative to international standards according to:

(2) $$\delta^{13}C\;\text{or}\;\delta^{15}N=\left(\frac{R_{\text{sample}}}{R_{\text{standard}}}-1\right)\times 1000,$$

where R represents the ratio of ^13^C/^12^C or ^15^N/^14^N. Analytical precision was < 0.15‰ for both carbon and nitrogen isotopes.

Calibration was performed using international reference materials of known isotopic composition. A laboratory working standard was analyzed alongside every batch of samples to assess analytical reproducibility, and measured values fell within the certified confidence intervals. In addition, eight blanks were analyzed at the beginning of each batch to control for potential contamination.

Because lipids are naturally depleted in ^13^C, variations in tissue lipid content can bias the interpretation of carbon isotope values. To assess and correct for this bias, we examined the C:N mass ratios of each sample, a reliable proxy for lipid content (Post et al. 2007). 106 samples with C:N ratios > 3.76 were considered to have elevated lipid content and were normalized using the lipid correction equation developed from deep‐sea fishes of the North Atlantic according to Hoffman and Sutton (2010):

(3) $$\delta^{13}C_{\text{protein}}=\delta^{13}C_{\text{bulk}}+\frac{-6.39\times \left(3.76-C:N_{\text{bulk}}\right)}{C:N_{\text{bulk}}}$$

Five samples with C:N ratios > 8 were not fitted for correction and were therefore removed from the analyzes. This correction ensured that $\delta^{13}$C values more accurately reflected ecological patterns rather than biochemical variation due to lipid composition in muscle tissue.

An integrated stable isotope framework was designed to disentangle the ecological drivers of isotopic variation, distinguish intraspecific from intergroup trophic variability and characterize patterns of resource partitioning within micronektonic fish assemblages.

The relationship between $\delta^{15}$N values and individual size was explored at the assemblage, functional group and species levels using linear models with coefficients of variation to assess the dispersion of values.

For each depth‐time strata sampled, we computed the median and standard deviation of $\delta^{15}$N and $\delta^{13}$C values per species to explore trophic variation at the species level, associated with their feeding guilds from the literature.

To investigate the relative contributions of different ecological and biological factors to isotopic variation, we performed a variance partitioning analysis using multiple linear regression models (Legendre and Legendre 2012). The analysis focused on $\delta^{15}$N values, an informative proxy for trophic structuring in this context. The influence of five predictor variables was tested: functional group, assemblage, sampling station, body size and species identity. The full model included all predictors, and models with each predictor alone or in pairs were used to estimate the proportion of variance explained by each variable and by shared effects. Unique contributions of each predictor were approximated by differences in adjusted $R^{2}$ between the full model and models including all other predictors, and the remaining variance was considered shared among predictors. ANOVA‐type permutation tests were performed for each model configuration to test the significance of the predictors' effects on $\delta^{15}$N.

To test whether $\delta^{15}$N values differ significantly between functional groups within each assemblage, we applied Kruskal–Wallis tests followed by Games‐Howell post hoc tests to accommodate unequal variances and sample sizes (R package rstatix). Functional groups with more than five samples in one assemblage were considered to allow for comparison. When significant, this analysis revealed which functional groups occupied distinct trophic positions within specific assemblages.

## 3 Results

### 3.1 Functional Diversity

**FIGURE 4:**
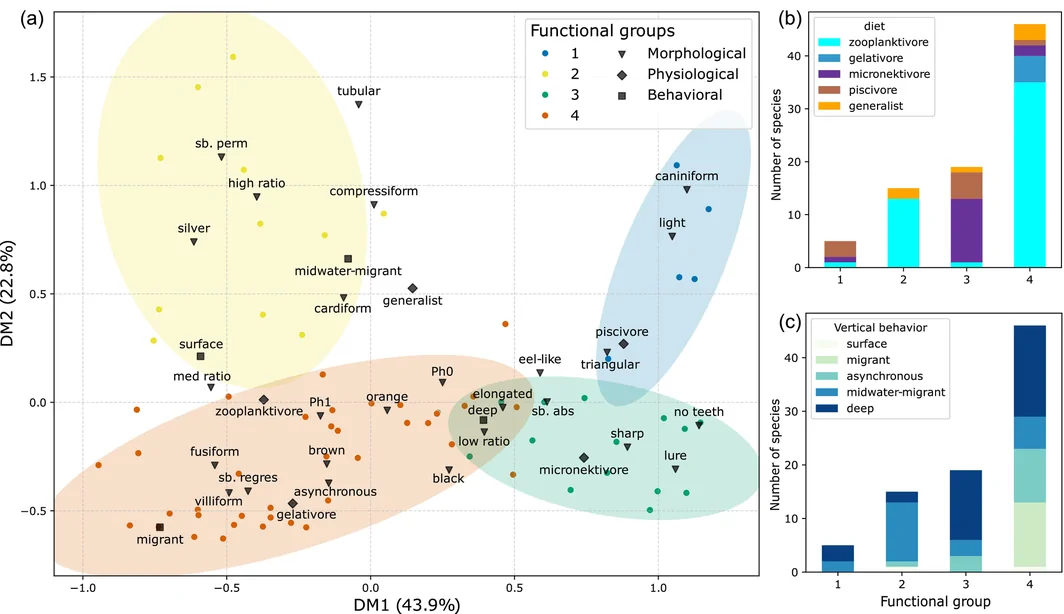
Biplot of the Multiple Component Analysis (with proportion of variance explained) showing the functional space in two dimensions (a). Each point corresponds to one functional entity (FE), and trait categories (text labels listed in Table 1) are projected in two dimensions based on the same multivariate space according to their association with FEs. Ellipses represent the dispersion of FEs belonging to the same functional group (FG). Right panels represent the distribution of trait categories among FGs for two traits including diet (b) and the vertical behavior (c).

The processes that structure the diversity of micronektonic fishes from the surface to 1300 m depth were investigated using a Multiple Component Analysis (MCA). Most of the 85 species were distributed across 75 functional entities (FEs), meaning that most of the species (66) had a unique combination of traits. FEs were categorized into 4 functional groups (FGs) based on their distance in the functional space (Figures 4, S4 and S3).

The functional space is represented by the first three dimensions of the MCA performed on categorical data of the trait matrix, which explained 77% of the total inertia. The first axis was positively correlated with the trophic position of species ($R^{2}$ = 0.61) and showed a weaker correlation with the maximum depth of occurrence ($R^{2}$ = 0.20), two traits that were reported in FishBase for each species. Two other traits characterized the second dimension and were associated with FG2: a permanent swim bladder and tubular eyes.

Zooplanktivores were predominantly located in the negative values of the first dimension, including FG2 and FG4. FG1 and FG3 mainly include micronektivores and piscivores with positive values in the first dimension. The absence of a swim bladder, sharp teeth and a fusiform body are the traits that contributed the most to the first dimension: they distinguish between FG3 and FG4.

Four functional groups were identified through clustering, each with the following characteristics:

- FG1 comprises 5 FEs composed of 5 species, all of the order Aulopiformes (Figure S5). These species are characterized by an elongated body of light color, caniniform teeth, no swim bladder and the presence of tubular eyes. They are mainly composed of piscivores that reside in the mesopelagic zone and are associated with a high trophic position.
- FG2 consists of 15 species divided into 11 FEs and mainly comprises Sternoptychidae. This functional group is characterized by zooplanktivorous fishes that are typically secondary consumers with a relatively low trophic position, a compressiform body with a silver color, cardiform teeth, a permanent swim bladder, the presence of tubular eyes with a high eye/body length ratio, and they are mainly composed of midwater migrants.
- FG3 comprises 17 FEs composed of 21 species mainly consisting of Stomiidae. This functional group is characterized by micronektivorous fish with a high trophic position, long and slender teeth, an elongated body with a black color and a low eye/body length ratio, the absence of a swim bladder, ventral photophores and the presence of a lure for five species. They mainly are deep pelagic fishes.
- FG4 has the most species, that is, 44 species from three families, distributed among 38 FEs and it mainly includes Myctophidae. FG4 is characterized by planktivorous fish with numerous needle‐like teeth, a fusiform body with a brown color, a regressive swim bladder and the presence of photophores. They perform all different kinds of vertical behavior, from surface migrant patterns to deep residents.

**FIGURE 5:**
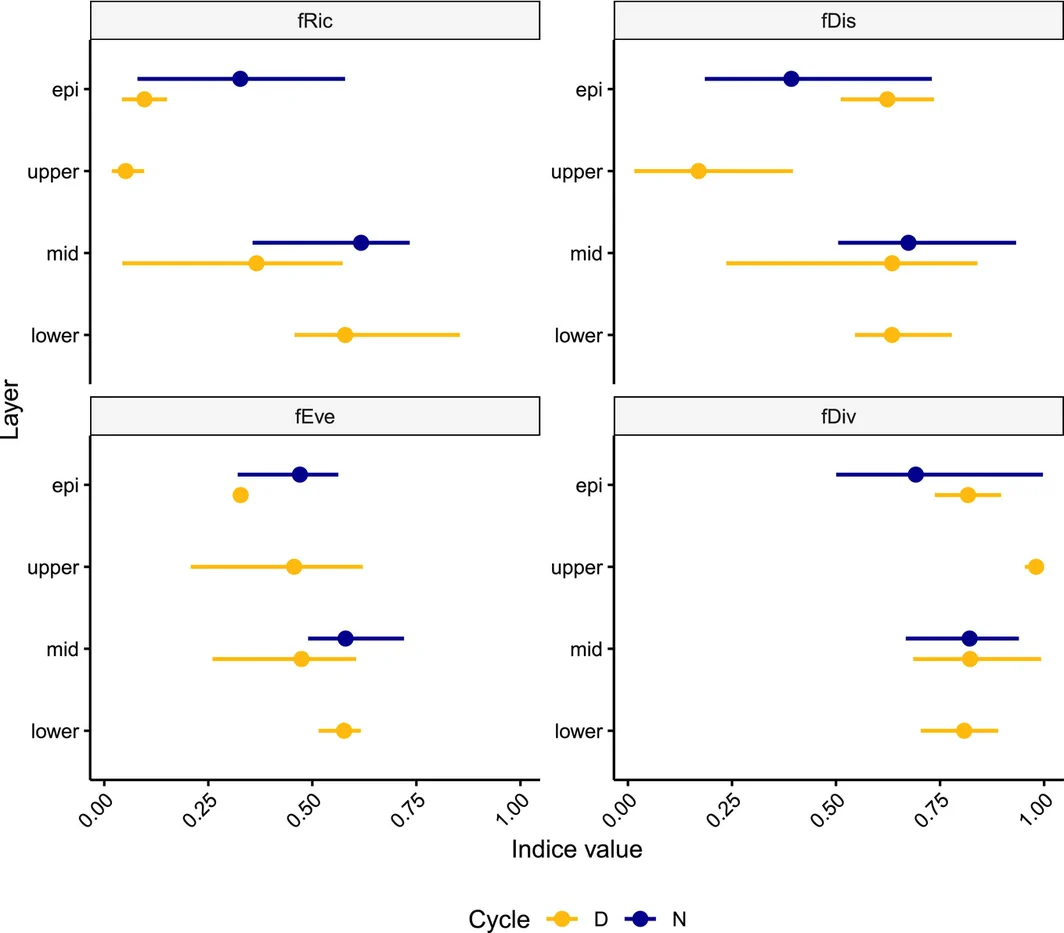
Functional indices computed for each depth layer including epipelagic (epi), upper‐mesopelagic (upper), mid‐mesopelagic (mid) and lower‐mesopelagic (lower), and during day (D) and night (N). Each panel represents the functional richness (fRic), functional evenness (fEve), functional dispersion (fDis) and functional divergence (fDiv). The dots represent the median, and the horizontal lines show the difference between the minimum and maximum values.

Functional indices were computed day and night for each depth layer (epipelagic, upper‐mesopelagic, mid‐mesopelagic and lower‐mesopelagic) based on the coordinates of FEs in the MCA and their abundance in the assemblages (Figure 5). The variability of these functional indices observed across stations was higher in the epipelagic zone during the night and in the mid‐mesopelagic layer both day and night. This suggests that the structure of these assemblages may be particularly influenced by environmental variations linked to the sampling station.

Functional richness (fRic), which represents the volume occupied by the assemblage in the functional space, was generally higher in the deeper layers (mid‐ and lower mesopelagic zones) than in the shallower epipelagic and upper mesopelagic layers. In layers sampled both day and night (epipelagic and mid‐mesopelagic), fRic was consistently higher at night, suggesting that the expansion of the functional space is due to diel vertical migrants coming from deeper layers (Figure 2).

Functional evenness (fEve) ranged around ∼0.5 both day and night and at all depth layers but showed a slight increasing trend with depth, ranging from 0.32 in the epipelagic layer during the day to 0.58 in the lower mesopelagic layer. fEve also increased at night in both the epipelagic and mid‐mesopelagic layers, indicating a more even distribution of the corresponding FEs.

Functional dispersion (fDis) was highly variable between assemblages. The lowest average value (0.17) was observed in the upper mesopelagic layer, indicating that FEs in this assemblage showed reduced dispersion in the functional space compared to other layers.

Functional divergence (fDiv) was > 0.6 in all depth layers, with the upper mesopelagic layer exhibiting the highest value (fDiv = 0.98), suggesting that abundant species in this assemblage were concentrated toward the edges of the functional space.

**FIGURE 6:**
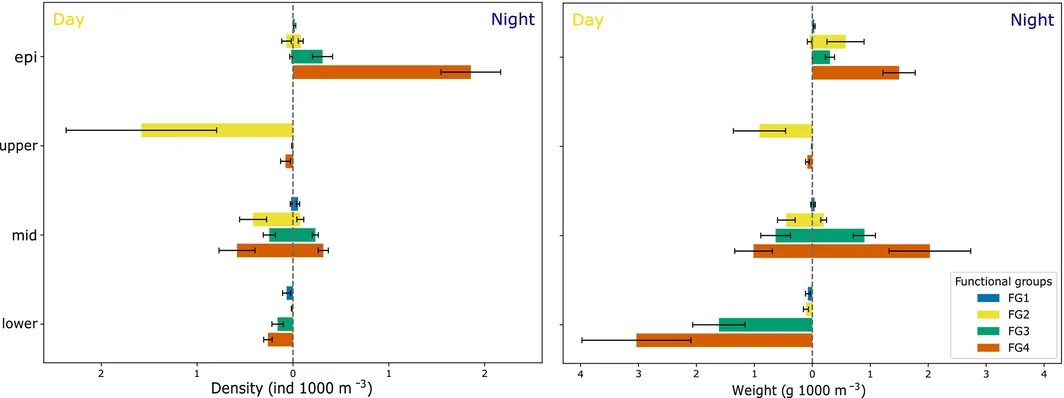
Distribution of functional groups by depth layers both day and night. The error bars correspond to the variability due to the differences observed between the stations.

The epipelagic layer had a very low micronektonic fish abundance during the day, while at night it had the highest abundance of all depth layers. It was primarily composed of species from FG4, with a smaller contribution from species in FG2 and FG3 (Figure 6). Species richness (SR, Figure S6) and functional richness (fRic) in the epipelagic layer were both very low during the day (SR = 5.5, fRic = 0.1) and increased at night (SR = 13.5, fRic = 0.4). In contrast, functional dispersion (fDis) was higher during the day, suggesting a greater ecological specialization among resident surface species. During the day, FEs were mostly located at the vertices of the functional space, with no single species dominating the assemblage. At night, the arrival of vertically migrating species led to a more functionally redundant community structure.

Abundance in the upper mesopelagic layer was predominantly composed of a single functional group (FG2). It exhibited the lowest specific richness, averaging 7.3 ± 3.4, along with the lowest functional richness (fRic = 0.05) and functional dispersion (fDis = 0.17), but the highest functional divergence (fDiv). This pattern can be attributed to the small number of FEs and their limited distribution within the functional space. The functional space was minimally occupied, with the most abundant species positioned on the vertices, primarily driven by the dominance of *Argyropelecus hemigymnus* .

The deeper layers (lower and mid) showed similar patterns, mainly composed of FG3 and FG4, with the presence of FG2 in the mid‐mesopelagic layer. Functional richness and dispersion were highly variable within the stations in the mid‐mesopelagic layer, mostly during the day. These layers displayed lower abundance than the upper ones but a significantly higher amount of biomass (Figure 6).

### 3.2 Trophic Structure

Stable isotope analyzes were conducted on individuals sampled across four distinct assemblages, including the epipelagic layer during the night (Epi‐Night), the mid‐mesopelagic layer during the day and night (Mid‐Day and Mid‐Night) and the lower‐mesopelagic layer during the day (Lower‐Day).

#### 3.2.1 Variance Partitioning of $\delta^{15}N$ Values

To assess the drivers of variation in trophic position, we tested the individual and independent effects of biological (body size), ecological (functional group) and environmental (station and assemblage) predictors on $\delta^{15}$N values. All predictors showed significant effects on $\delta^{15}$N values, including functional group (F=15.6, p<0.001), assemblage (F=12.0, p<0.001), and body size (F=19.6, p<0.001). Station also had a weaker but still significant effect on $\delta^{15}$N values (F=2.6, p=0.01).

Variance partitioning of $\delta^{15}$N values revealed the effects of independent predictors, including station (5.3%), body size (8.6%), assemblage (14.2%) and functional group (18.0%). Overall, the model explained 39% of the total variance of $\delta^{15}$N, with 10% of the estimated shared variance attributed to these predictors. When species level was used instead of functional group, the model explained 83.5% of the variance, with 71.1% uniquely attributed to species and 7% to shared variance. These results indicate that species level is the main driver of $\delta^{15}$N values, while functional groups remain important, although some groups are confined to particular assemblages or size classes.

Variance partitioning was conducted at the functional group level to identify the main factors influencing variability in $\delta^{15}$N, including species level, assemblage and body size (Table 2). Species level emerged as the dominant explanatory factor across all functional groups. Assemblage also significantly contributed to isotopic variation, with $R^{2}$ values ranging from 0.14 to 0.42. Body size had a weaker overall influence but still contributed significantly to the variation of $\delta^{15}$N values in FG3.

**TABLE 2:** Proportion of variance in $\delta^{15}$N explained within each functional group (FG), including the number of assemblages, samples and species (*N*).

FG	$N_{\text{samples}}$	$N_{\text{species}}$	$N_{\text{assemb}.}$	$R_{\text{size}}^{2}$	$R_{\text{assemb}.}^{2}$	$R_{\text{species}}^{2}$	Total
1	11	3	4	0.03	0.42	0.89	0.97
2	32	9	3	—	0.16	0.57	0.64
3	40	7	4	0.18	0.14	0.43	0.64
4	118	24	4	0.05	0.23	0.68	0.64

*Note:* Variance is partitioned by size, assemblage, species identity ($R^{2}$) and by the full model (Total).

At the species level, body size showed a more consistent effect (Table 3). Among the 12 species tested (each with at least eight samples), nine exhibited a positive correlation between body size and $\delta^{15}$N values. The remaining three species, characterized by broader size distributions, did not show a clear size effect on $\delta^{15}$N isotope values.

**TABLE 3:** Results of linear regression at species level between $\delta^{15}$N values and body size associated to the corresponding functional group (FG), size range in mm and the number of samples (*N*).

Species	FG	Size	*N*	Slope	$R^{2}$	*p*
*Acrtozenus risso*	1	88.5–155.0	8	—	—	0.28
*Argyropelecus olfersii*	2	38.2–80.9	11	—	—	0.09
*Argyropelecus hemigymnus*	2	31.5–36.4	8	0.28	0.68	< 0.05
*Stomias boa*	3	81.7–300.0	15	0.01	0.56	< 0.01
*Chauliodus sloani*	3	110.0–240.0	13	0.01	0.40	< 0.05
*Malacosteus niger*	3	102.2–177.0	8	—	—	0.63
*Benthosema glaciale*	4	40.6–78.3	30	0.14	0.56	< 0.001
*Lobianchia gemellarii*	4	58.4–86.8	12	0.08	0.88	< 0.001
*Lampanyctus macdonaldi*	4	125.9–149.9	8	—	—	0.94
*Lampanyctus crocodilus*	4	79.9–145.6	8	0.07	0.69	< 0.05
*Bathylagus euryops*	4	134.7–185.0	8	—	—	0.85
*Cyclothone* sp.	4	35.0–64.7	16	0.21	0.71	< 0.001

#### 3.2.2 Depth Layers Structure and Stable Isotope Ratios

The $\delta^{15}$N values showed a significant increase with depth between the Lower‐Day and each of the shallower assemblages, but the difference was not significant between Epi‐Night and the mid‐mesopelagic assemblages during day and night (Figure S7).

**FIGURE 7:**
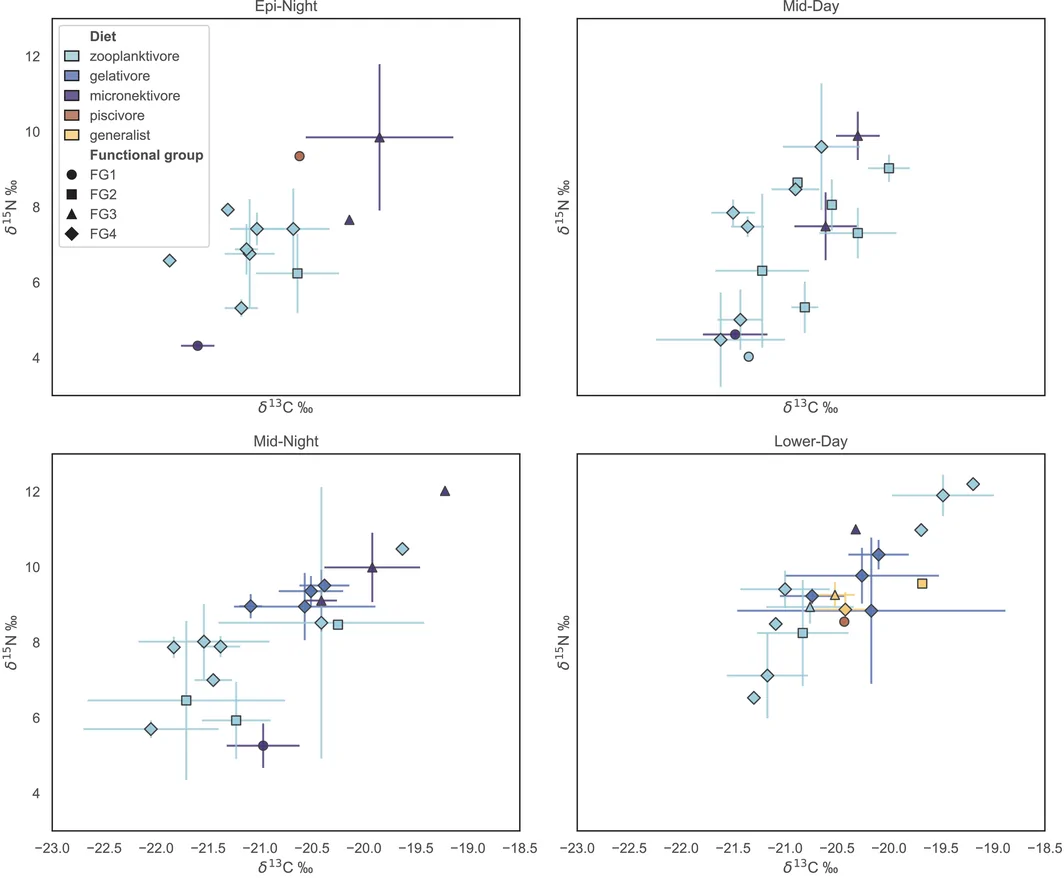
Bivariate plots of $\delta^{13}$C and $\delta^{15}$N values (‰) for species by assemblages including epipelagic during night (Epi‐Night), mid‐mesopelagic during day and night (Mid‐Day and Mid‐Night) and lower‐mesopelagic during day (Lower‐Day), and functional groups (FG). Diamonds indicate median values; error bars represent standard deviations.

Stable isotope composition varied among the trophic guilds and depth‐time strata (Figure 7). Zooplanktivores, which comprised the highest species richness, abundance and number of isotopic samples, exhibited the widest range of $\delta^{15}$N and $\delta^{13}$C values, particularly in the deeper mesopelagic layers (Lower‐Day). They are mainly represented by FG2 and FG4, occupying similar $\delta^{15}$N ranges across depth layers, although FG2 exhibited higher $\delta^{13}$C values than FG4. Micronektivores from FG3 and piscivores occupied the upper end of the $\delta^{15}$N gradient, whereas micronektivores from FG1 displayed the lowest isotopic values, likely reflecting the predominance of juvenile individuals. Generalists, which were only present in the Lower‐Day strata, showed intermediate $\delta^{15}$N and $\delta^{13}$C values. In Epi‐Night strata, trophic guilds and functional groups were clearly separated along the $\delta^{15}$N axis, with limited overlap between zooplanktivores and higher trophic groups. In contrast, isotopic niches became progressively more compressed in the Mid‐Day and Mid‐Night strata, where overlap among trophic guilds increased despite the persistence of a trophic gradient. The Lower‐Day strata exhibited the highest overall $\delta^{15}$N and $\delta^{13}$C values and the greatest isotopic overlap among trophic guilds, particularly between micronektivores, piscivores and generalists.

**FIGURE 8:**
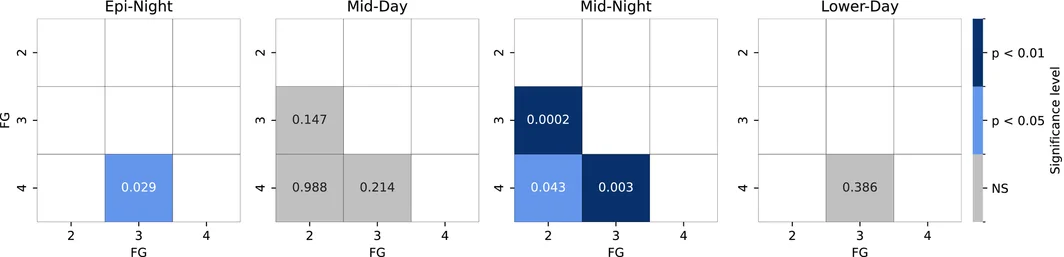
Games‐Howell post hoc significance results between $\delta^{15}$N values of functional groups by depth‐time strata.

Kruskal–Wallis tests revealed significant differences in $\delta^{15}$N values between functional groups only in Epi‐Night (p=0.01) and Mid‐Night (p=0.0001) layers, but they were not significant in Mid‐Day and Lower‐Day. In the Epi‐Night layer, FG4 was the dominant group by abundance (∼2 ind 1000 $m^{-3}$) and by the number of samples, including 27 samples from 7 species. This group displayed distinct isotopic values compared to FG3 (Figure 8), the second most represented group in Epi‐Night, which included only two species in the isotope and trawl samples, dominated by *Stomias boa* and the presence of *Chauliodus sloani* . These micronektivorous species exhibited higher values of both $\delta^{15}$N and $\delta^{13}$C than the other species in FG4 composed of zooplanktivores (Figure 7).

In the Mid‐Day layer, FG2, FG3 and FG4 were the most abundant (∼0.2–0.5 ind 1000 $m^{-3}$ for each functional group), and showed no significant difference in $\delta^{15}$N values (Figure 8). The Mid‐Night layer, composed mainly of FG2, FG3 and FG4 as well, showed significant differences in isotopic values between FGs (p<0.001), where $\delta^{15}$N values differed significantly between all the functional groups. In the lower‐mesopelagic layer during the day (Lower‐Day), dominated by FG3 and FG4, $\delta^{15}$N values were not significantly different between these groups.

## 4 Discussion

Our study combined a community sampling of 85 species with behavioral, morphological and trophic traits, together with stable isotope analyzes. The vertical resolution of the sampling design, based on acoustically defined depth layers, provided a characterization of functional diversity and isotopic niches, which allows functional strategies to be directly related to trophic organization in the mesopelagic zone.

### 4.1 Trade‐Offs Among Traits in Micronektonic Fishes

**FIGURE 9:**
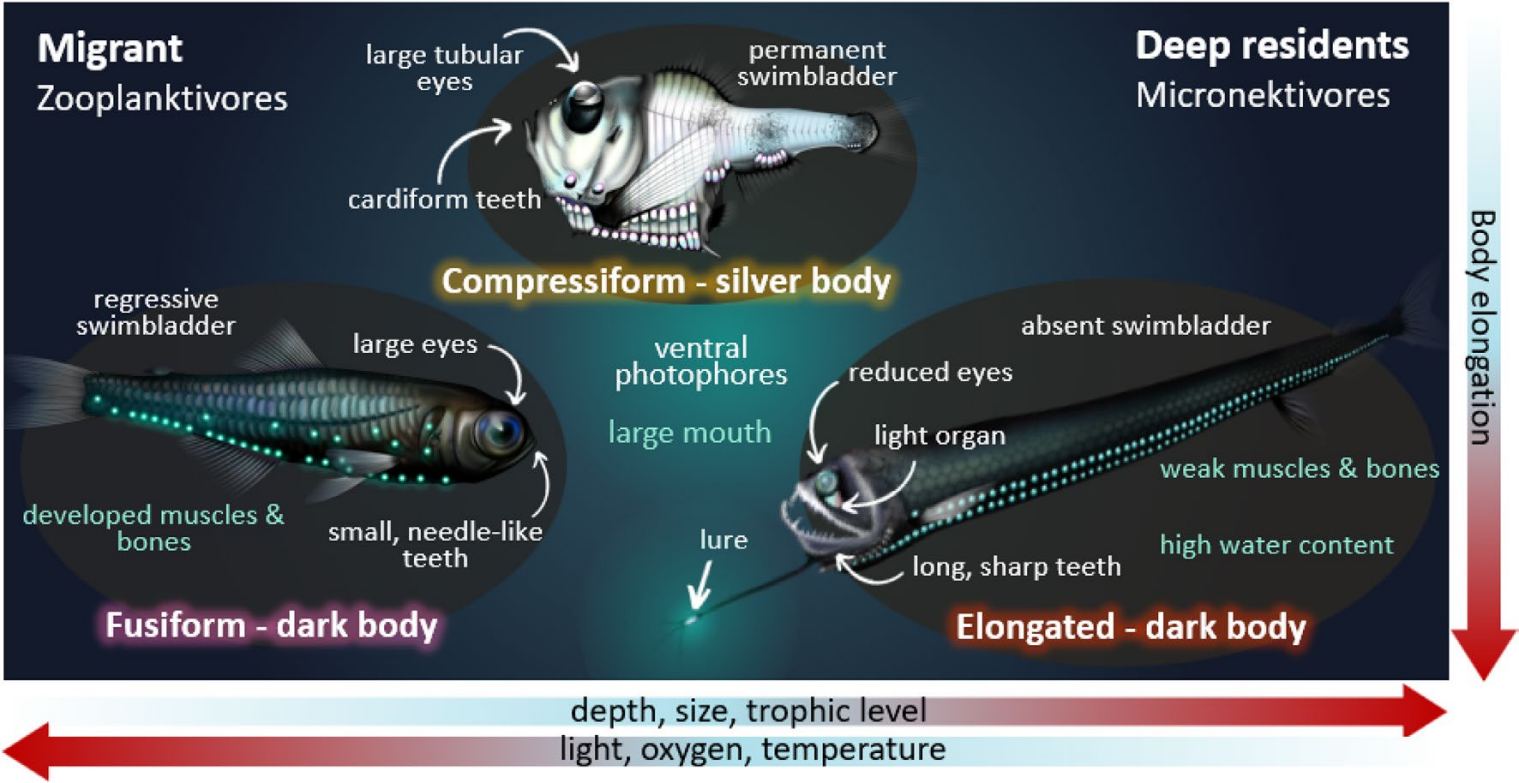
Key trade‐offs observed in mesopelagic fishes. This illustrates three distinct functional groups based on common species including FG4 (left), FG2 (middle) and FG3 (right). Fish illustrations are from Tom Langbehn, licensed under CC BY 4.0.

Micronektonic fish species were classified into four functional groups, each defined by distinct combinations of morphological and ecological traits (Figure 9). The resulting functional space emphasized trade‐offs among resource acquisition, survival strategies and environmental adaptations. Functional differentiation was primarily driven by trophic position, with negative values on the first axis associated with zooplanktivorous species and positive values associated with piscivorous and micronektivorous species (Figures 4 and 7).

The dominance of one functional group (FG4) across all depth layers, combined with its high species richness and strong representation of diel vertical migrants, suggests that FG4 plays a pivotal role in mesopelagic ecosystem functioning. Its widespread occurrence along the water column reflects a high degree of ecological flexibility, enabling species, with a high dominance of zooplanktivores, to exploit resources across contrasting environmental conditions and contribute substantially to vertical connectivity and energy transfer. Half of the species in this group perform either surface or asynchronous migrations and share key traits, including villiform teeth, a regressive (sometimes non‐functional or lipid‐filled) swim bladder, a fusiform body shape and the presence of photophores (Figure 4, Figure S4). These characteristics support efficient swimming and active foraging of zooplankton (Marshall 1971; De Busserolles et al. 2020), with a diversity of preys and depth distribution. However, species of this functional group occurring at greater depths exhibit some trait differentiations. This includes darker pigmentation, more elongated body forms, reduced or absent bioluminescence and a fully regressed swim bladder. These features are consistent with adaptations to deeper low‐light environments with limited prey–predator interactions, where crypsis, hydrodynamic efficiency and lipid‐based buoyancy reduce energetic costs (Neighbors and Nafpaktitis 1982; Seibel and Drazen 2007; Neat and Campbell 2013). Ontogenetic increases in body size, are accompanied by shifts in vertical distribution and trophic position (Table 3), although the observed increase in bulk $\delta^{15}$N with depth may also reflect depth‐related changes in isotopic baselines associated with microbial reworking and organic matter remineralization (Gloeckler et al. 2018).

Species that inhabit intermediate depths within the upper mesopelagic zone at night (i.e., midwater migrants) were clustered in FG2, corresponding to negative values on the first axis and positive values on the second axis (Figure 4). These species, including hatchetfish, often exhibit silvery coloration and large ventral photophores, consistent with counter‐illumination strategies to avoid predation (Andresen et al. 2025). In Sternoptychidae (6 out of 15 species in FG2), which are strongly laterally compressed, the body surface closely approximates an ideal vertical mirror, maximizing reflectivity and minimizing visual detection from below (Priede 2017). Some species also possess tubular eyes (*Argyropelecus* sp.) or spherical eyes with a pronounced ventral area of retinae (*Sternoptyx* sp.), providing good upward visual resolution specialized in detecting the prey silhouettes against dim background light (Priede 2017). These traits appear to be adapted to the relatively shallow mesopelagic zone, where sufficient daylight allows predators to detect prey. In this environment, their trade‐offs enhance camouflage but reduce swimming capacity, thereby limiting the extent of diel vertical migration observed for these species.

Deep‐resident species were predominantly associated with FG1 and FG3 (Figure 4c) and exhibited morphological and physiological traits typical of adaptations to aphotic high‐pressure environments. These included elongated bodies, reduced eyes and the absence of a swim bladder (Figure 4a). Species were primarily micronektivores and piscivores, with a well developed dentition. Notably in FG3, a subset of six (out of 21) species in this group possessed a bioluminescent lure, an adaptation typical of ambush nektivores that use light to attract prey in the dark (Gartner Jr et al. 1997). At these depths, visual systems are adapted for detecting point‐source bioluminescence rather than forming detailed images, a key adaptation to extreme low‐light conditions (De Busserolles et al. 2020). In contrast, FG1 also comprises piscivores deep‐resident species, but is distinguished from FG3 by a light color body, a different type of dentition and the presence a tubular eyes for some species (Figure S4). These features are typically associated with a more visually oriented predation strategy, particularly for detecting silhouettes or point‐source light from prey above (Warrant et al. 2003; De Busserolles et al. 2020).

### 4.2 Vertical Structuring of Micronektonic Fish Communities

The ecological structure of micronektonic fish communities was examined across distinct depth layers during the day and night. Specimens collected in targeted trawl deployments enabled the characterization of functional diversity within these depth‐time strata, revealing contrasting patterns between the epipelagic zone and the lower mesopelagic layers. In addition, stable isotope analysis provided further information on the trophic structure of these communities, linking functional composition with patterns of resource partitioning along the vertical gradient.

Generally, all depth layers showed a high level of functional divergence (fDiv), being higher where lower functional richness occurred (Figure 5). High fDiv indicates strong niche differentiation among dominant species, implying reduced interspecific competition for resources (Mouillot et al. 2005; Farré et al. 2016). Consequently, mesopelagic communities exhibiting high fDiv may promote enhanced ecosystem functioning through more efficient resource use. In addition, our results showed that functional richness is higher in the lower parts of the mesopelagic zone, reflecting greater species specialization. This finding suggests that this stable and nutritionally poor environment with low interactions promotes functional and taxonomic diversity (Figure 5, Figure S6). The observed pattern aligns with ecological competition theory, which predicts that under food‐limited conditions, species tend to specialize and minimize interspecific dietary overlap to reduce competition (Schoener 1974; Abrams 1983), resulting in high specialization of deep pelagic fish species (Loutrage et al. 2024). This diversity of body adaptations in deep‐sea fishes has been linked to elevated evolutionary rates, particularly in locomotor traits, reflecting relaxed selective pressures on certain aspects of locomotor performance compared to shallow‐water environments (Martinez et al. 2021).

The epipelagic layers are primarily inhabited by vertically migrating organisms that ascend to feed on nutrient‐rich surface waters during the night. Within this depth layer, FG4 was dominant in terms of abundance and biomass, while functional richness and dispersion were relatively low (Figures 5 and 6). This pattern, combined with overlapping and restricted stable isotope values in FG4 (Figure 7), suggests high functional redundancy and shared dependence on similar food resources (Hopkins and Gartner 1992; Finke and Snyder 2008). The pronounced temporal overlap in feeding activity, restricted mainly to nighttime, likely intensifies competition. However, the high productivity of surface waters at night and potential fine‐scale resource partitioning may buffer competitive exclusion, allowing coexistence despite apparent niche similarity. FG4 was dominated by Myctophidae, the most diverse and abundant family of mesopelagic fishes that we found. Despite their morphological similarities, many species within this family can coexist thanks to niche partitioning along multiple ecological axes, including specific diet type, vertical habitat and feeding chronology (Schoener 1974; Eduardo et al. 2021; Loutrage et al. 2024; García‐Seoane et al. 2025).

Deeper mesopelagic layers showed the highest functional richness, with high species richness and lower abundance (Figures 5 and 6, Figure S6). Biomass remained important even at low abundance due to larger‐bodied organisms (Figure 6). Stable isotope data ($\delta^{15}$N) showed higher values in lower mesopelagic layers than in shallower layers (Figure S7), consistent with higher trophic positions and changes in the isotopic baseline with depth (Hannides et al. 2013). Although deep mesopelagic food webs may rely on multiple organic matter sources, with greater reliance on sinking and suspended particles (Gloeckler et al. 2018; Choy et al. 2015), bulk $\delta^{15}$N values alone cannot distinguish among these basal resources. At the same time, strong isotopic redundancy was observed in mid and lower mesopelagic layers, as shown by the marked overlap in isotopic niches among functional groups FG2, FG3 and FG4 (Figures 7 and 8). This suggests that despite trait divergence, these groups occupy similar isotopic space. Nevertheless, the lower mesopelagic layers encompass a greater diversity of feeding guilds. In these interaction‐limited environments, high functional diversity might reflect the exploitation of a broader range of prey and feeding strategies, potentially supported by multiple organic matter pathways.

### 4.3 Limitations

Regarding stable isotope analysis, samples were collected based on an opportunistic sampling strategy, where the number of individuals analyzed per species was primarily determined by their availability in the trawl hauls. Consequently, dominant or more frequently caught species are overrepresented in the isotopic dataset. While this approach ensures sufficient sample sizes for the most ecologically relevant species, it may underrepresent rarer species and limit comparative analyzes across the full community. Nonetheless, it provides robust trophic insights into the most influential components of the assemblage.

An additional limitation relates to potential sampling biases associated with net avoidance behavior. Many mesopelagic fish are capable of detecting and avoiding approaching trawls, which can lead to underestimation of their abundance and biomass in net samples. Avoidance behavior depends on species swimming ability, size and morphology and therefore may bias species composition and size structure in catches, particularly in deep hauls. Furthermore, mesopelagic organisms can respond to artificial light from vessels by moving away or changing depth, which may further reduce catch efficiency or alter the vertical distribution sampled (Kaartvedt et al. 2012). Consequently, highly mobile or visually sensitive species, including migratory taxa, may be underrepresented in trawl samples, potentially influencing the observed functional structure and vertical distribution patterns, particularly in lower mesopelagic layers considering the time of net removal.

Another limitation of our analysis is the reliance on midwater trawl samples, which are known to underestimate small, fragile and elongated species such as *Cyclothone* spp. These fishes are among the most abundant vertebrates as mesopelagic resident species and contribute substantially to mesopelagic biomass and trophic transfer (Nelson et al. 2016; Sarmiento‐Lezcano et al. 2022). This bias may result in an incomplete picture of mesopelagic fishes' functional diversity, as *Cyclothone* spp. and other small micronekton fill important ecological roles, acting as prey for higher trophic levels and mediating carbon flux through diel vertical migrations. Incorporating alternative sampling methods (e.g., BIONESS multinets) will be necessary to better quantify the contribution of these taxa and to obtain a more comprehensive understanding of mesopelagic fish community functioning.

Regarding the functional analysis, our selected traits encompassed a range of ecological functions; yet, many relevant functional traits remain unexplored. The choice was constrained by data availability, particularly for rare species in the mesopelagic zone. Consequently, the FGs identified here, as well as the trait space covered, depend directly on the species pool studied and their selected traits. Rarer species collected only once during the survey were excluded from the analysis. While their ecological roles may not match those of the more abundant taxa considered, it remains an open question whether including additional rare species or phylogenetic clades would significantly alter the distribution of FGs and functional diversity, especially in deeper layers where rarer forms might fill unique ecological niches.

### 4.4 Perspectives

Our results indicate that vertical connectivity in the mesopelagic zone is primarily driven by diel vertical migrants that link surface and deep layers, while functional diversity at depth is shaped by a range of resident deep‐dwelling strategies. This functional structuring provides important insights for improving the representation of mesopelagic communities in global biogeochemical models, which often overlook ecological complexity at these depths. Explicitly distinguishing between migratory and resident strategies, and incorporating key functional traits such as body size, feeding guild, predation mode and migration behavior, could enhance predictions of trophic pathways, energy transfer and carbon export within models of the biological carbon pump and micronekton dynamics. Advancing our understanding of mesopelagic ecosystems will require further investigation into the dependence of mesopelagic fishes and their prey on marine snow.

From a biogeochemical perspective, the coexistence of actively migrating species and deep‐dwelling species that primarily occupy midwater layers supports a dual role of mesopelagic fauna in the biological carbon pump. Migratory organisms drive active carbon flux through diel vertical migration, whereas deep‐dwelling species contribute more to carbon transformation, retention and recycling within the mesopelagic zone (e.g., Longhurst 1985; Robinson et al. 2010; Davison et al. 2013). This functional arrangement refines the classical Vinogradov ladder concept by emphasizing functionally distinct pathways and depth‐specific roles. Future studies could benefit from better constraining isotopic baselines in the mesopelagic zone, where microbial reworking and organic matter remineralization can complicate the interpretation of bulk $\delta^{15}$N values. Compound‐specific isotope analyzes of amino acids would help distinguish trophic position from baseline variation and improve the identification of carbon pathways supporting deep‐pelagic food webs.

Our trait‐based analyzes reveal distinct ecological functions in the mesopelagic zone that vary across depth strata, with important implications for ecosystem resilience under environmental change. Deep‐dwelling micronektonic fishes exhibit low abundance but relatively high species and functional richness, associated with high functional richness, suggesting limited functional redundancy and increased vulnerability affecting key deep‐sea functional processes, including a diversity of feeding strategies. These patterns confirm previous observations of high trophic and functional specialization in mesopelagic fish communities across different oceanic regions (Kumar et al. 2017; Aparecido et al. 2023; Loutrage et al. 2025). These findings suggest that common ecological structures may govern the functional diversity across different pelagic systems, where scarce resources lead to trait divergence to limit competition. This counteracts earlier studies suggesting that the low availability of food resources at depth would constrain the trophic and morphological specialization of species (Childress and Meek 1973; Ebeling and Cailliet 1974).

To deepen our understanding of the processes structuring micronektonic fish communities, future research should explore how species' functional traits, particularly metabolic rates, influence vertical distribution and resource use, especially in low‐oxygen or dim‐light environments. Comparative studies across latitudinal and environmental gradients could reveal how temperature, oxygen, nutrient concentrations and light shape depth preferences, migratory behavior and functional group composition. Extending trait‐based analyzes beyond fishes to include decapods, amphipods, cephalopods and gelatinous taxa will provide a more comprehensive view of functional overlap, competition and ecological interactions in the midwater ecosystem (Hopkins and Sutton 1998; Loutrage et al. 2025). Integrating functional traits, trophic interactions and biogeochemical processes will enable a more accurate linking of mesopelagic community structure to ecosystem functioning and carbon cycling in this globally important yet understudied realm.

## 5 Conclusions

To address the key questions raised in the introduction regarding vertical structuring, functional diversity, trophic functioning and coexistence of mesopelagic communities, our results provided a trait‐based perspective on how micronektonic fishes are organized along the water column:

- How are mesopelagic communities structured along the water column? Mesopelagic communities are vertically structured into distinct functional assemblages associated with feeding guilds and body size. Shallow layers are dominated by migratory zooplanktivores that connect surface and mesopelagic depths, whereas deeper layers are increasingly composed of resident taxa adapted to stable, low‐light environments and occupying higher trophic positions, including micronekton feeders as well as zooplanktivores with a greater reliance on gelatinous prey and marine snow. This pattern reflects a transition from dynamic, vertically connected communities from epipelagic to mid‐mesopelagic layers (0 to ~700 m) to more stratified and functionally differentiated assemblages at greater depths (~700 to 1300 m).
- Which key traits and trade‐offs explain the processes driving functional diversity and their link with the vertical dimension? Functional diversity is driven by trade‐offs among feeding guilds (e.g., zooplanktivory and micronektivory) that differ in their environmental adaptations and foraging strategies. These trade‐offs arise from constraints imposed by the vertical environment, where energy acquisition, predator avoidance and physiological tolerance to pressure and light conditions shape distinct adaptive strategies. They are primarily expressed through morphological traits such as body shape, dentition and swim bladder type. As individuals grow, ontogenetic shifts in morphology and metabolism are often accompanied by increases in trophic position and a transition to deeper habitats. This pattern is particularly evident in FG4, the most widespread functional group, whose species exhibit adaptations spanning a broad depth range and display considerable variation in vertical distribution and isotopic values.
- How do stable isotope values reflect trophic and functional redundancy or complementarity? Stable isotope values ($\delta^{15}$N) indicate increasing trophic positions with size and depth but also reveal substantial isotopic niche overlap among deeper specimens, pointing to trophic redundancy. However, this apparent redundancy likely masks underlying complementarity, as groups with similar isotopic signatures may exploit different prey types or feeding pathways, as suggested by the different feeding guilds and the functional diversity.
- How is species coexistence shaped by vertical gradients within the pelagic environment? Species coexistence is shaped by vertical gradients through a combination of habitat partitioning and functional differentiation. In upper layers, coexistence is facilitated by vertical dispersion and resource variability, whereas in deeper, interaction‐limited environments, it relies more on subtle niche partitioning, supported by functional diversity and the use of distinct carbon sources.

Overall, our study deepens our understanding of the functioning of micronektonic fish communities by linking functional diversity with vertical distribution and resource partitioning. This integrated approach highlights how ecological trade‐offs associated with resource acquisition and environmental adaptation shape the functional organization and coexistence of species. Improving the representation of these processes is essential for predicting how global change and biodiversity loss may affect deep‐sea ecosystem functioning (Martin et al. 2020).

## Author Contributions

**Hélène Thibault:** conceptualization (lead), data curation (supporting), formal analysis (lead), funding acquisition (supporting), investigation (lead), methodology (lead), resources (equal), visualization (lead), writing – original draft (lead). **Frédéric Ménard:** conceptualization (supporting), data curation (supporting), funding acquisition (supporting), investigation (supporting), methodology (supporting), project administration (supporting), supervision (lead), validation (equal), writing – original draft (supporting), writing – review and editing (supporting). **Gildas Roudaut:** data curation (supporting), investigation (supporting), methodology (supporting), resources (supporting), validation (supporting), visualization (supporting), writing – review and editing (supporting). **Anne Lebourges‐Dhaussy:** data curation (supporting), investigation (supporting), methodology (supporting), resources (supporting), validation (supporting), writing – review and editing (supporting). **Yves Cherel:** data curation (supporting), investigation (supporting), methodology (supporting), resources (supporting), validation (supporting), writing – review and editing (supporting). **Alejandro Ariza:** methodology (supporting), resources (supporting), validation (supporting), visualization (supporting), writing – review and editing (supporting). **Leandro Nolé Eduardo:** data curation (supporting), methodology (supporting), validation (supporting), writing – review and editing (supporting). **Séverine Martini:** conceptualization (supporting), data curation (lead), funding acquisition (supporting), investigation (supporting), methodology (supporting), project administration (supporting), resources (supporting), supervision (lead), validation (supporting), visualization (supporting), writing – original draft (supporting), writing – review and editing (supporting).

## Funding

APERO project, National Research Agency under the grant APERO, Grant/Award Number: ANR ANR‐21‐CE01‐0027, and by the French LEFE‐Cyber program (CNRS, INSU).

## Conflicts of Interest

The authors declare no conflicts of interest.

## Supporting information


**Figure S1:** Classification and delimitation of sounds scattering layers (SSL) for each stations based on detection by an EK80 operating at 18 and 38 kHz.
**Figure S2:** Flowchart describing the main steps of the functional analysis (see text). The abundance matrix is composed of functional entities (FE) densities across assemblages. The trait matrix is composed of a set of functional trait for each FE. Functional richness (fRic), functional evenness (fEve), functional dispersion (fDis) and functional divergence (fDiv) are functional indices computed from the abundance matrix and the functional space (see text).
**Figure S3:** Silhouette score as a function of the number of functional groups. The peak value indicates the optimal number of clusters.
**Figure S4:** Classification of species based on MCA distance from categorical traits including main body color (color), body shape (body), eye shape (eye), teeth pattern (teeth), type of diet (diet), presence of bioluminescent organ (biolu), type of swim bladder (swimbladder), migration pattern (migration) and class of ratio between eye diameter and standard length (eye/TL class). Trait categories are referred in Table 1.
**Figure S5:** Taxonomic composition of the functional groups.
**Figure S6:** Barplot of diversity metric (top panels) including species richness (SR), functional entities richness (FE) and Shannon index of each assemblage. Functional space in two dimensions (bottom panels) based on the MCA coordinates for each trawling station.
**Figure S7:** Boxplots of $\delta^{15}$N values distribution by assemblages including epipelagic layer at night (Epi‐Night), mid‐mesopelagic layer during day and night (Mid‐Day and Mid‐Night) and lower‐mesopelagic layer sampled during day (Lower‐Day). A Games Howell test was performed to test the significant differences between the assemblages (**: *p* ≤ 0.01; ****: *p* ≤ 0.0001).
**Table S1:** Summary of trawl sampling stations.
**Table S2:** Summary analysis of stable isotopes by species. Values are presented as mean ± variance with the number of samples per species (*N*).

## Data Availability

The acoustic dataset supporting this study is publicly available from SEANOE: Roudaut Gildas, Lebourges‐Dhaussy Anne, Guidi Lionel, Memery Laurent (2023). Acoustic data from APERO cruise on R/V Thalassa. https://doi.org/10.17882/103238.
