# Supplementary material for: Functional and Trophic Structuring of Micronektonic Fishes in the Northeast Atlantic
Source: Ecol Evol. 2026 Sep 10;16(9):e74275. doi: 10.1002/ece3.74275 (PMC13561858; doi:10.1002/ece3.74275)
Supplement: Supplementary file 1 [file ECE3-16-e74275-s001.pdf]

Supporting Information

5.1 | Sampling strategy

Depth layers were defined for each station based on acoustic detections (Fig.S1). Since the frequency response varies with depth and can therefore differ for the same migrating organism depending on its position in the water column throughout the day, two acoustic frequencies (18 and 38 kHz) were used to identify sound scattering layers. The epipelagic layers were defined between 0-200 m for all stations. Deeper layers were defined according to variations in acoustic detections between day and night, which allowed for consistency across the stations. The lower limits of the upper mesopelagic layer correspond to Sv38>Sv18 by night and the mid-mesopelagic layer to Sv18>Sv38 by night.

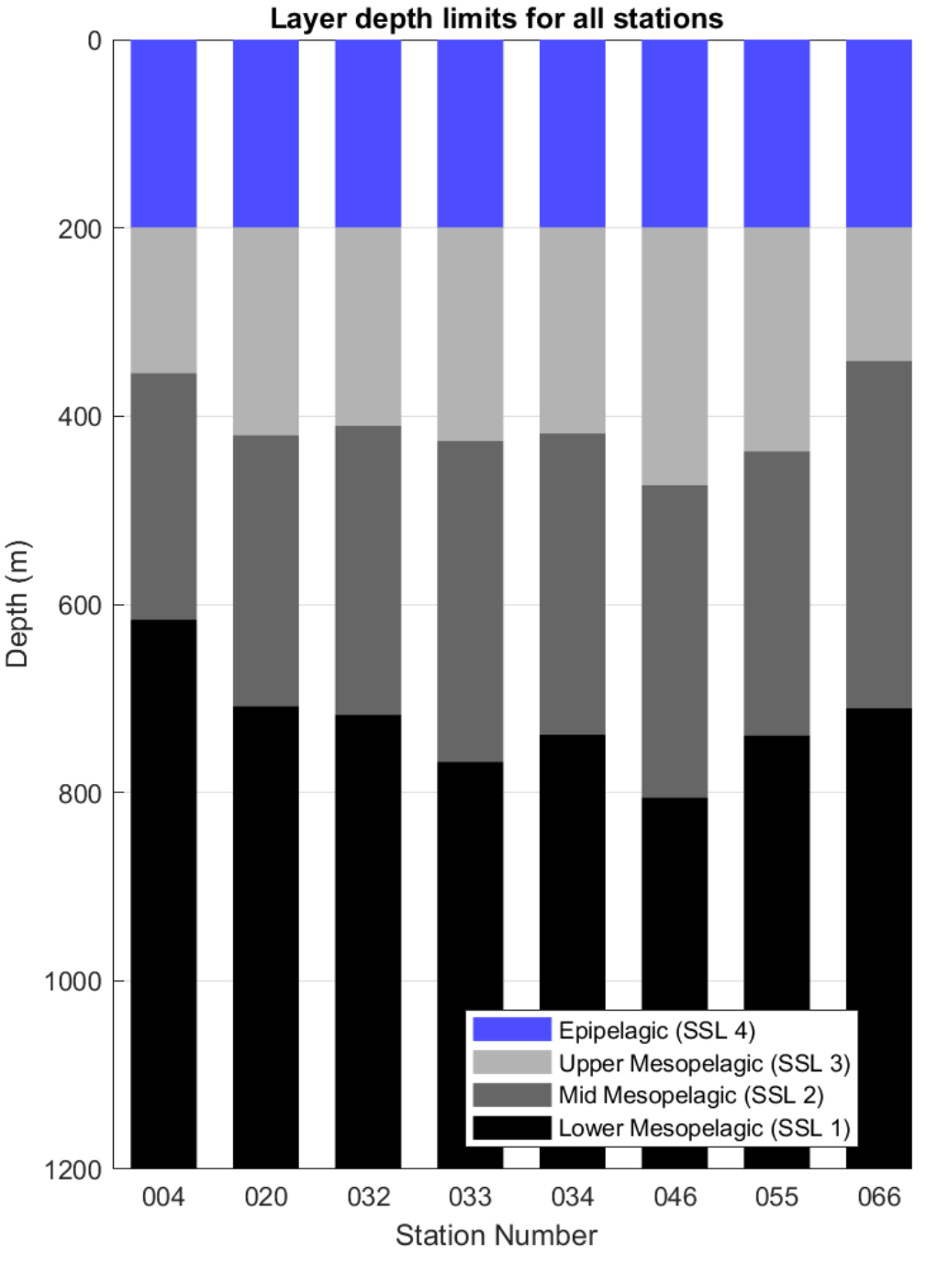

FIGURE S1 Classification and delimitation of sounds scattering layers (SSL) for each stations based on detection by an EK80 operating at 18 and 38 kHz.

TABLE S1 Summary of trawl sampling stations.

Station	Date and time	Depth-time strata	Target depth (m)
S04	06-07 22:50	Mid-Night	570
S04	06-08 01:00	Epi-Night	0-160
S04	06-08 14:50	Mid-Day	380
S20	06-12 10:45	Mid-Day	500
S20	06-12 23:20	Mid-Night	480
S20	06-13 01:10	Epi-Night	25
S20	06-13 13:55	Lower-Day	700-1100
S32	06-16 23:10	Mid-Night	470-510
S32	06-17 01:45	Epi-Night	0-80
S32	06-17 15:10	Mid-Day	500
S32	06-17 17:20	Lower-Day	750
S33	06-18 23:20	Mid-Night	435-620
S33	06-19 01:20	Epi-Night	20-200
S33	06-19 09:55	Mid-Day	500-600
S33	06-19 15:45	Lower-Day	800-1300
S34	06-20 23:20	Mid-Night	415-530
S34	06-21 00:50	Epi-Night	20-30
S34	06-21 09:30	Mid-Day	340-530
S34	06-21 17:10	Epi-Day	150-295
S55	07-01 14:10	Mid-Day	585-600
S55	07-01 15:55	Epi-Day	140
S55	07-01 18:20	Upper-Day	400
S55	07-01 22:40	Mid-Night	590
S55	07-02 00:35	Epi-Night	20-75
S66	07-05 12:10	Lower-Day	700-800
S66	07-05 16:20	Upper-Day	200-240
S66	07-06 17:20	Mid-Day	400-600
S66	07-06 22:50	Mid-Night	400-600
S66	07-07 00:30	Epi-Night	20-25

5.2 | Functional analysis

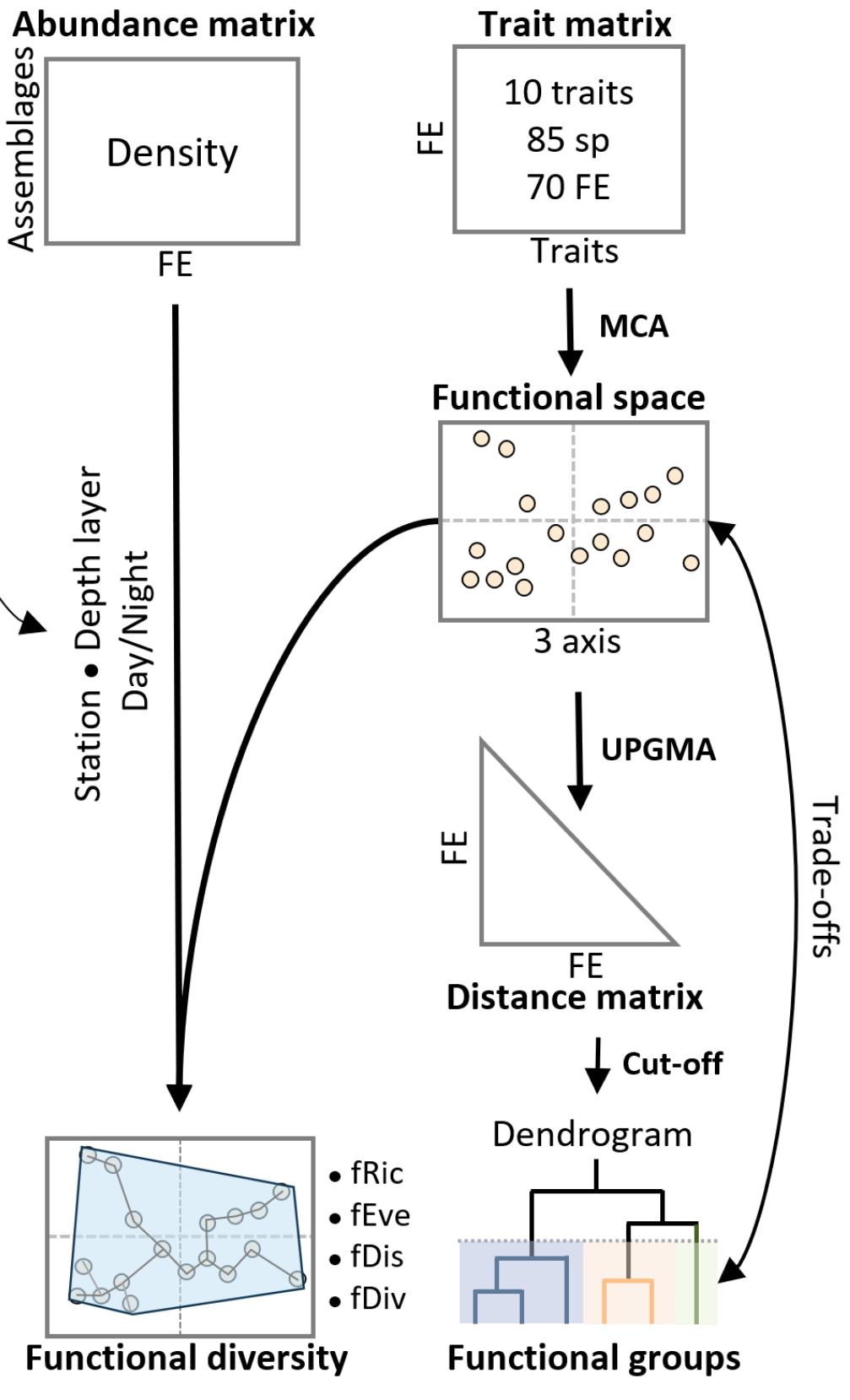

FIGURE S2 Flowchart describing the main steps of the functional analysis (see text). The abundance matrix is composed of functional entities (FE) densities across assemblages. The trait matrix is composed of a set of functional trait for each FE. Functional richness (fRic), functional evenness (fEve), functional dispersion (fDis) and functional divergence (fDiv) are functional indices computed from the abundance matrix and the functional space (see text).

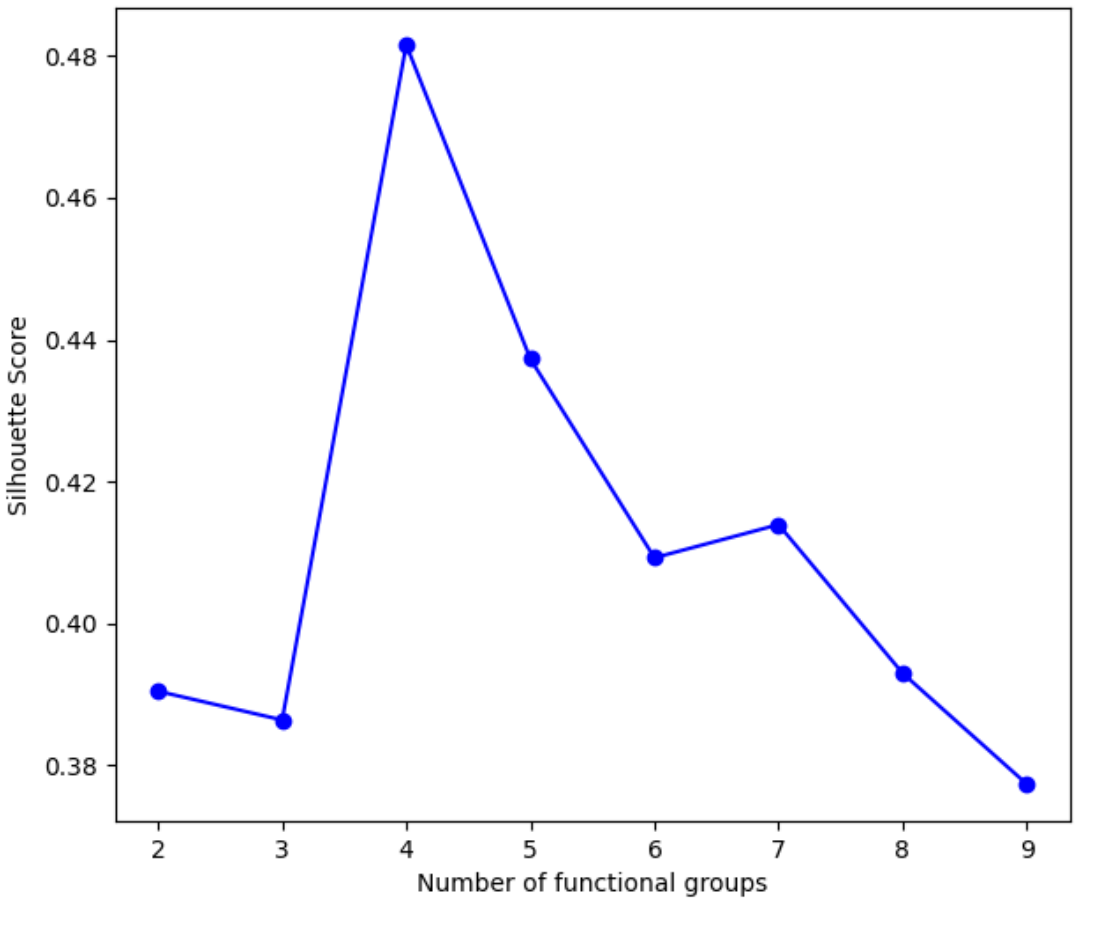

FIGURE S3 Silhouette score as a function of the number of functional groups. The peak value indicates the optimal number of clusters.

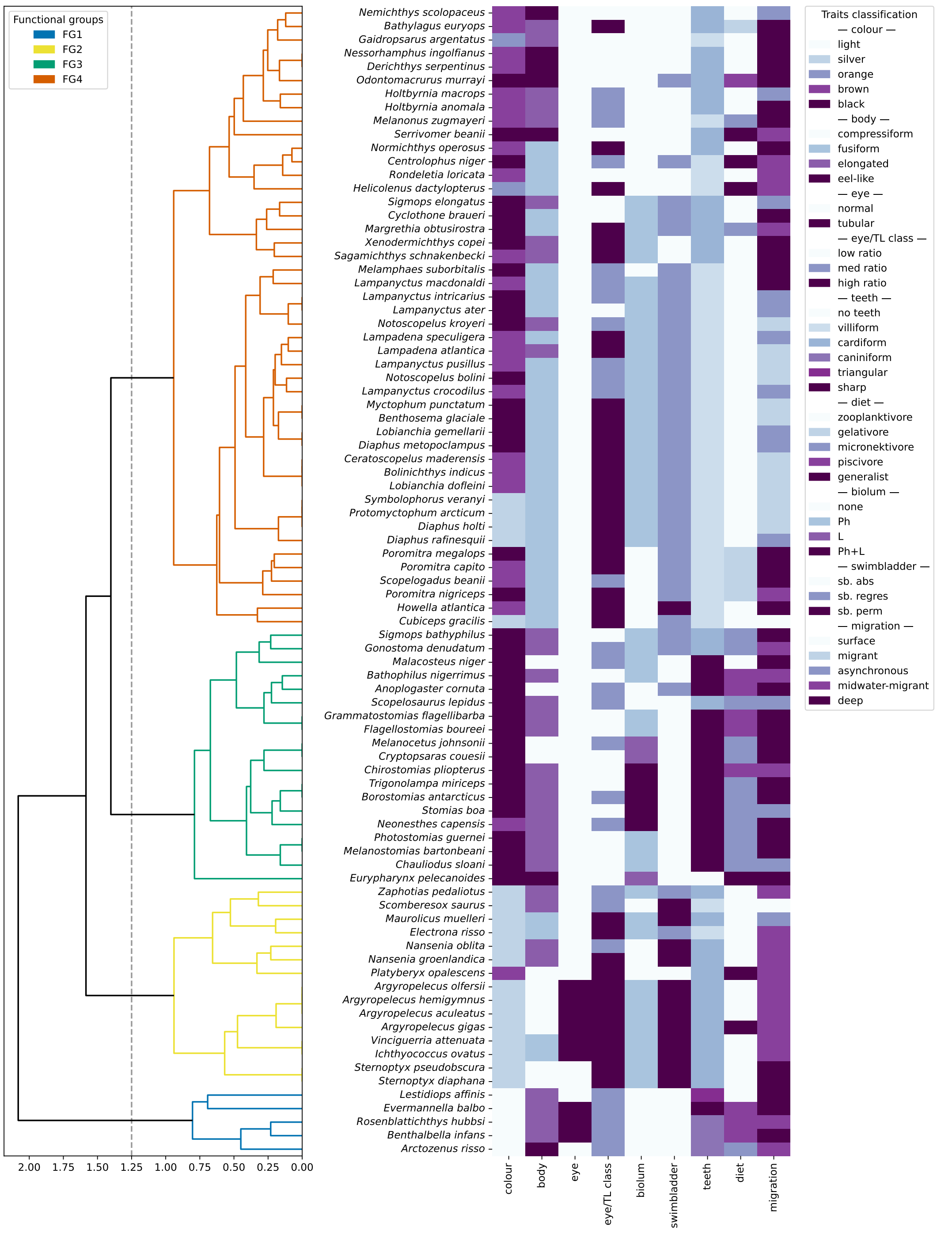

FIGURE S4 Classification of species based on MCA distance from categorical traits including main body colour (colour), body shape (body), eye shape (eye), teeth pattern (teeth), type of diet (diet), presence of bioluminescent organ (biolu), type of swim bladder (swimbladder), migration pattern (migration) and class of ratio between eye diameter and standard length (eye/TL class). Trait categories are referred in Table 1.

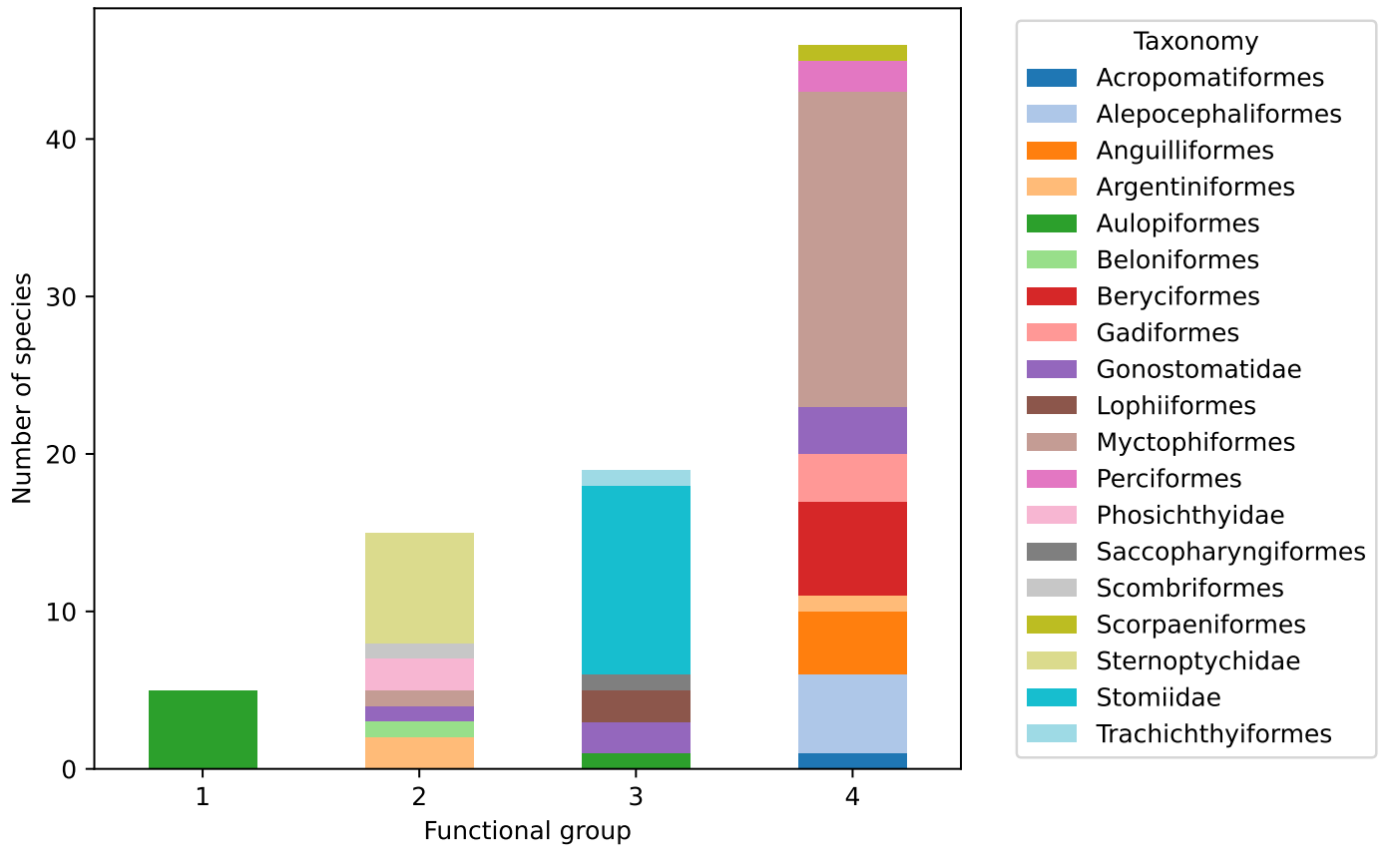

FIGURE S5 Taxonomic composition of the functional groups.

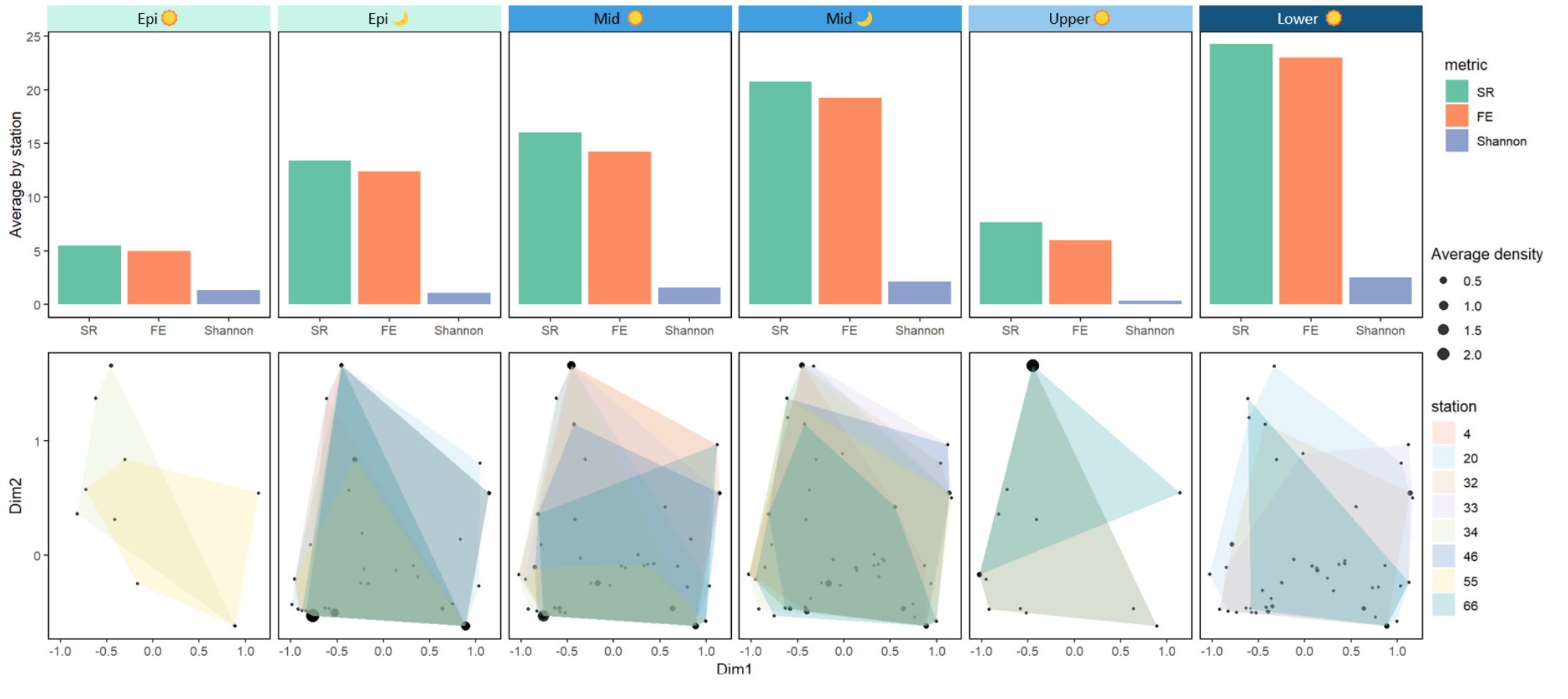

FIGURE S6 Barplot of diversity metric (top panels) including species richness (SR), functional entities richness (FE) and Shannon index of each assemblage. Functional space in two dimensions (bottom panels) based on the MCA coordinates for each trawling station.

5.3 | Stable isotopes

TABLE S2 Summary analysis of stable isotopes by species. Values are presented as mean ± variance with the number of samples per species (N).

Species	N	Size (mm)	$\delta^{13}C_{bulk}$	$\delta^{13}C_{corr}$	$\delta^{15}N_{bulk}$	C:N
Arctozenus risso	8	88.5-155.0	-21.32±0.19	-21.88±0.07	4.98±0.46	3.47
Argyropelecus aculeatus	4	27.7-39.5	-20.85±0.01	-21.59±0.02	5.30±0.18	3.37
Argyropelecus hemigymnus	8	31.5-36.4	-20.26±0.08	-21.04±0.07	7.78±0.34	3.35
Argyropelecus olfersii	11	38.2-80.9	-20.92±0.38	-21.79±0.36	6.38±2.88	3.31
Bathylagus euryops	8	134.7-185.0	-21.02±1.08	-20.67±0.31	8.98±1.12	4.08
Benthalbella infans	2	121.6-135.3	-20.90±0.05	-20.53±0.02	8.95±0.33	4.0
Benthodesmus elongatus	1	238.0	-20.81	-21.55	7.33	3.37
Benthosema glaciale	30	40.6-78.3	-21.62±0.79	-20.95±0.20	8.48±2.71	4.37
Bolinichthys indicus	1	39.1	-22.11	-21.87	6.58	3.91
Borostomias antarcticus	7	211.0-312.0	-22.05±0.41	-19.70±0.23	11.39±0.13	6.07
Chauliodus sloani	13	110.0-240.0	-21.58±1.80	-20.52±0.18	8.40±0.99	4.97
Cubiceps gracilis	7	98.5-105.8	-21.92±0.43	-21.40±0.13	7.20±1.28	4.17
Cyclothone sp.	16	35.0-64.7	-20.59±0.71	-20.76±0.70	8.43±8.47	3.68
Diaphus holti	3	37.4-50.5	-21.91±0.27	-21.91±0.63	6.63±2.62	3.77
Diaphus metopoclampus	6	53.0-57.2	-21.74±0.19	-21.80±0.12	7.95±0.18	3.74
Diaphus rafinesquii	6	59.2-67.5	-21.49±0.08	-21.53±0.02	7.33±0.12	3.74
Dolicholagus longirostris	2	113.6-114.0	-23.71±0.03	-20.72±0.01	4.91±0.05	7.07
Electrona risso	6	46.9-65.5	-20.94±0.55	-21.32±0.31	7.78±2.12	3.55
Eurypharynx pelecanoides	6	293.0-414.0	-20.39±0.05	-20.87±0.10	9.45±0.27	3.5
Gonostoma denudatum	4	114.4-117.4	-20.30±0.04	-21.11±0.02	9.89±0.41	3.34
Howella atlantica	5	49.9-79.6	-21.39±0.36	-21.35±0.45	9.33±0.24	3.92
Lampadena speculigera	2	67.2-67.6	-20.96±0.23	-21.78±0.25	7.79±3.19	3.33
Lampanyctus ater	1	117.2	-21.73	-19.69	10.98	5.53
Lampanyctus crocodilus	8	79.9-145.6	-20.18±1.18	-21.05±0.99	8.10±5.39	3.31
Lampanyctus macdonaldi	8	125.9-149.9	-21.94±0.30	-19.67±0.24	11.85±0.30	5.93
Lampanyctus sp.	1	47.7	-21.93	-21.72	5.37	3.89
Lestidiops affinis	1	159.0	-21.35	-22.04	4.03	3.4
Lobianchia dofleini	1	39.2	-22.58	-21.31	7.93	4.7
Lobianchia gemellarii	12	58.4-86.8	-20.74±0.49	-21.17±0.29	8.93±0.88	3.53
Malacosteus niger	8	103.2-177.0	-21.33±0.13	-20.75±0.23	8.92±0.20	4.19
Melanomus zugmayeri	1	150.8	-19.78	-20.67	9.29	3.3
Melanostomias bartonbeani	3	198.0-276.0	-21.06±2.85	-19.72±0.42	10.72±1.34	5.14
Myctophum punctatum	5	79.0-97.8	-21.44±0.10	-20.98±0.05	7.18±0.20	4.06
Nanobrachium atrum	1	138.0	-21.93	-20.46	11.59	4.87
Nansenia oblita	3	125.9-129.8	-23.74±0.08	-20.49±0.15	6.72±1.24	7.66
Normichthys operosus	2	113.6-135.8	-19.41±0.10	-20.08±0.61	11.34±1.49	3.41
Notoscopelus kroyeri	6	38.0-147.0	-21.07±0.37	-21.05±0.16	8.00±3.31	3.78
Notoscopelus sp.	2	35.9-36.8	-21.35±0.00	-21.80±0.00	5.71±0.04	3.51

Paralepididae	2	59.6-62.4	-20.63±0.03	-21.18±0.02	6.55±0.00	3.46
Platyberyx opalescens	1	119.4	-19.68	-20.60	9.56	3.29
Poromitra capito	5	75.7-81.5	-21.12±0.30	-21.00±0.03	9.14±0.07	3.85
Poromitra megalops	4	42.8-64.0	-20.24±0.08	-20.97±0.13	9.92±0.28	3.38
Scopelogadus beanii	6	84.8-113.2	-20.55±0.48	-20.92±0.09	9.43±0.28	3.6
Serrivomer beanii	6	490.0-695.0	-20.37±0.10	-21.19±0.09	9.13±0.71	3.33
Sigmops elongatus	2	153.0-170.0	-21.06±0.10	-21.21±0.02	7.15±0.69	3.67
Sternoptyx diaphana	5	27.8-37.2	-20.82±0.39	-21.46±0.28	7.79±0.98	3.42
Sternoptyx pseudobscura	5	32.1-38.9	-21.13±0.21	-21.32±0.14	8.12±2.38	3.66
Stomias boa	15	81.7-300.0	-20.37±0.83	-20.63±0.24	9.43±2.29	3.68
Symbolophorus veranyi	6	67.2-108.6	-21.64±0.20	-21.90±0.41	6.11±0.99	3.68
Xenodermichthys copei	4	91.4-140.7	-21.38±0.04	-22.04±0.00	7.82±0.08	3.41
Zaphotias pedaliotus	5	54.4-61.6	-19.99±0.04	-20.46±0.02	8.96±0.13	3.5

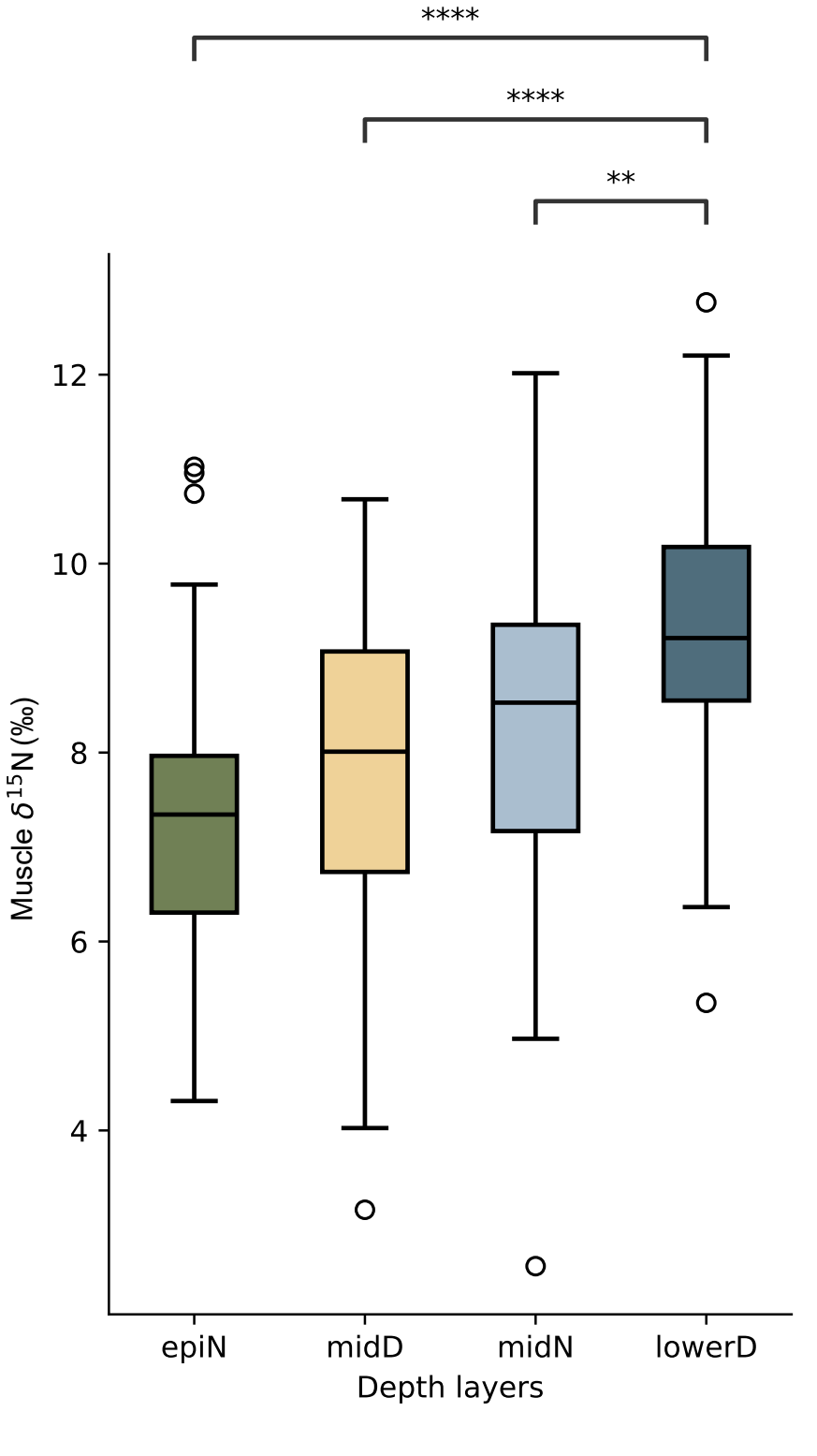

FIGURE S7 Boxplots of δ^{15}N values distribution by assemblages including epipelagic layer at night (Epi-Night), mid-mesopelagic layer during day and night (Mid-Day and Mid-Night) and lower-mesopelagic layer sampled during day (Lower-Day). A Games Howell test was performed to test the significant differences between the assemblages (**: p≤0.01; ****: p≤0.0001).
